# *Pestalotiopsis* and allied genera from *Camellia*, with description of 11 new species from China

**DOI:** 10.1038/s41598-017-00972-5

**Published:** 2017-04-13

**Authors:** Fang Liu, Lingwei Hou, Mubashar Raza, Lei Cai

**Affiliations:** 1 Chinese Academy of Sciences, Institute of Microbiology, State Key Laboratory of Mycology, Beijing, 100101 China; 2 University of Chinese Academy of Sciences, Beijing, 100049 China

## Abstract

A total of 124 *Pestalotiopsis-*like isolates associated with symptomatic and asymptomatic tissues of *Camellia sinensis* and other *Camellia* spp. from eight provinces in China were investigated. Based on single- and multi-locus (ITS, TEF, TUB2) phylogenies, as well as morphological characters, host associations and geographical distributions, they were classified into at least 19 species in three genera, i.e. *Neopestalotiopsis*, *Pestalotiopsis* and *Pseudopestalotiopsis*. Eight novel species in *Pestalotiopsis* and three novel species in *Pseudopestalotiopsis* were described. Our data suggested that the currently widely used loci in *Pestalotiopsis*-like genera do not consistently provide stable and sufficient resolution tree topologies, especially for *Neopestalotiopsis*. Moreover, the number, branch pattern and length of the conidial basal appendages were revealed to be phylogenetically informative characters in *Pestalotiopsis*.

## Introduction

*Camellia sinensis* (L.) Kuntze, widely grown in the tropics and subtropics, are commonly processed to produce popular beverage—tea. Many other species of *Camellia* are commercially important ornamentals, such as *C. japonica*, *C. oleifera* and *C. sasanqua*. According to the USDA database, 520 fungal species have been known to occur on *Camellia* spp., of which 303 were from *C. sinensis* ^1^. In our national wide investigation of fungi on *C. sinensis* in China, we have identified several dominant genera, namely *Colletotrichum* ^2^, *Diaporthe* ^3^ and *Pestalotiopsis*. While several studies documented *Pestalotiopsis* species occurring on tea planHowever, in the gene trees of Neopestalotiopsits^4–7^.

Some *Pestalotiopsis* species are known to cause diseases of foliage, stems and roots and cause considerable reduction on commercial production^5, 6, 8, 9^. For instance, grey blight disease of tea plants caused by *Pestalotiopsis* spp. resulted in 17% production loss in southern India^8^ and 10–20% yield loss in Japan^9^.

Traditionally taxonomy of *Pestalotiopsis* was limited to naming species according to the host from which they were first observed and the conidial morphologies such as colour intensities of the median conidial cell^10, 11^. With the introduction of multi-locus DNA sequence analyses, the traditional host-based classification system and the division of the groups and species based on conidial characters proved to be unreliable^12–18^. The use of molecular data in resolving *Pestalotiopsis* species was recently reviewed by Maharachchikumbura *et al*.^18^, and two new genera were established, i.e. *Neopestalotiopsis* and *Pseudopestalotiopsis* (*Ps*.). Up to now, seven species in *Pseudopestalotiopsis* and 25 in *Neopestalotiopsis* have been introduced^4, 17–19^, and 52 species in *Pestalotiopsis* have been phylogenetic verified^2, 17, 18, 20–22^. Among above, only four were associated with *C. sinensis*, i.e. *P. camelliae*, *P. furcate*, *Ps. ignota*, *Ps. theae* ^4–6^.

The current study aimed to investigate the taxonomic and phylogenetic diversity of *Pestalotiopsis-*like species associated with *C. sinensis* and other *Camellia* spp. based on morphology and molecular phylogenetic data. Eleven new species are described, illustrated and compared with allied specie.

## Results

### Phylogenetic species delimitation

#### *Neopestalotiopsis*

**Figure 1:**
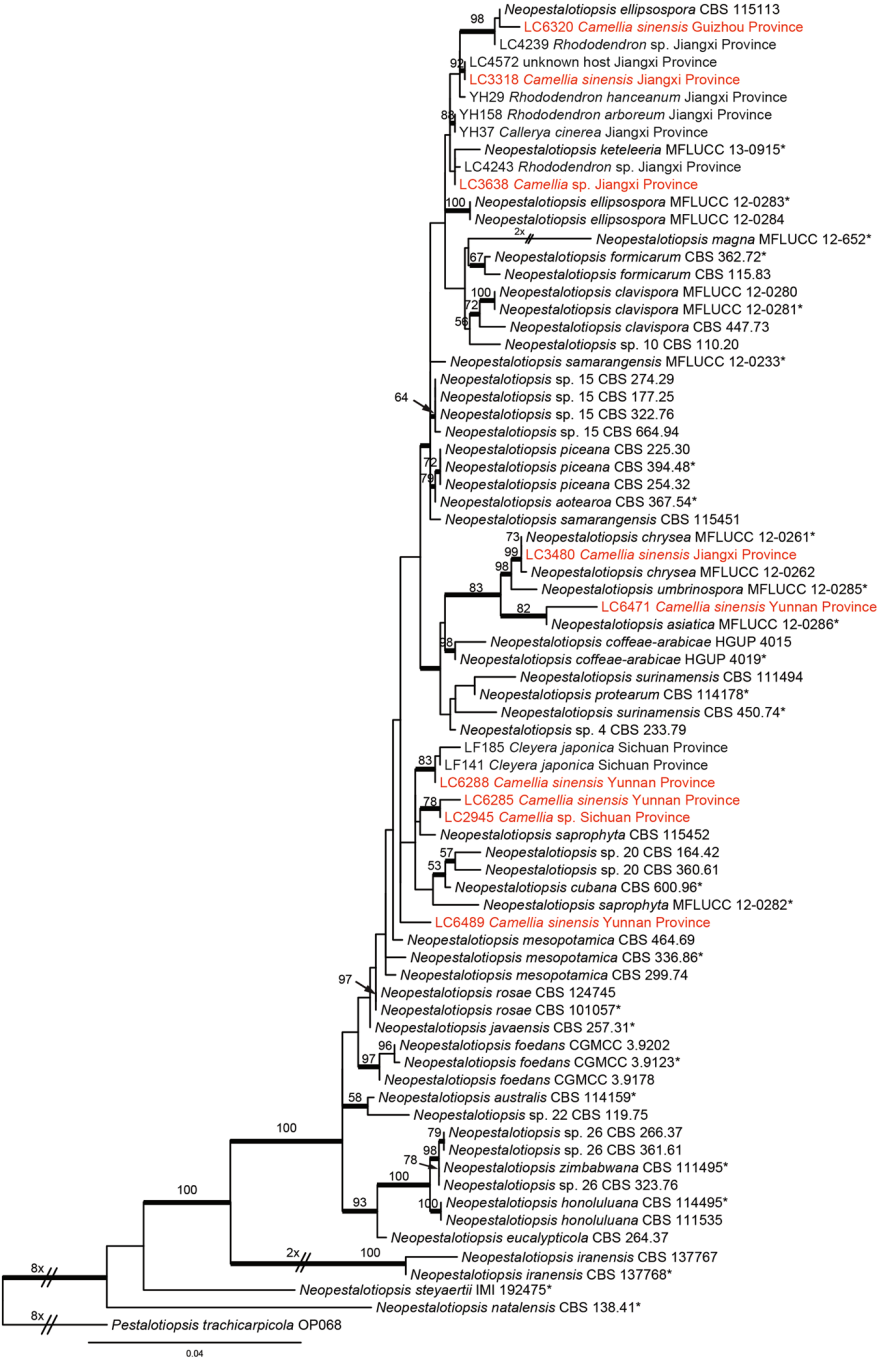
Phylogenetic tree of *Neopestalotiopsis* calculated with maximum likelihood analysis on a combined dataset of three-locus sequences (ITS, TUB2, TEF) by running RAxML v.7.0.3. The RAxML bootstrap support values (>50%) are displayed at the nodes. Thickened branches indicate branches also present in the Bayesian tree with >0.95 posterior probabilities. Stars indicate ex-type cultures. Strains from *Camellia* spp. in this study are in red colour.

Phylogenetic analyses of 101 isolates of *Neopestalotiopsis* were performed on single locus and concatenated datasets (ITS, TUB2, TEF), with *Pestalotiopsis trachicarpicola* (OP068) as outgroup (Fig. 1, Supplementary Figs S1–3). Because the length of published sequences obtained from NCBI varied significantly, the ragged sequences in the front and end of the alignments were cut off. The dataset used for phylogenetic analyses contained 444 characters with alignment gaps for ITS, 499 for TEF, and 412 for TUB2. For the Bayesian inference, a GTR+G model with gamma-distributed rate was selected for ITS and TUB2, and HKY+G model with gamma-distributed rate for TEF. The maximum likelihood tree confirmed the tree topology of the Bayesian consensus tree.

Phylogenetic gene trees conflicted with each other. Several clades representing known species on the concatenated gene tree were poorly supported on the single gene trees, with very short branches. Our result also demonstrated discordance between the three-gene (ITS, TUB2, TEF) tree (Fig. 1) and that inferred from Maharachchikumbura *et al*. (2014b, in Fig. 4). For example, two isolates of *N. samarangensis* in Maharachchikumbura *et al*.^18^, CBS 115451 and MFLUCC 12-0233, were separated into different clades in Fig. 1. This scenario also happened to *N. ellipsospora*, *N. mesopotamica*, *N. saprophyta*, and *N. surinamensis*, which implied that the topology of the 3-gene tree was unstable and the previously constructed backbone tree may not necessarily reflect the natural evolutionary relationships. *Neopestalotiopsis* isolates associated with *C. sinensis* and other *Camellia* spp. are therefore not classified in this study.

#### *Pestalotiopsis*

**Figure 2:**
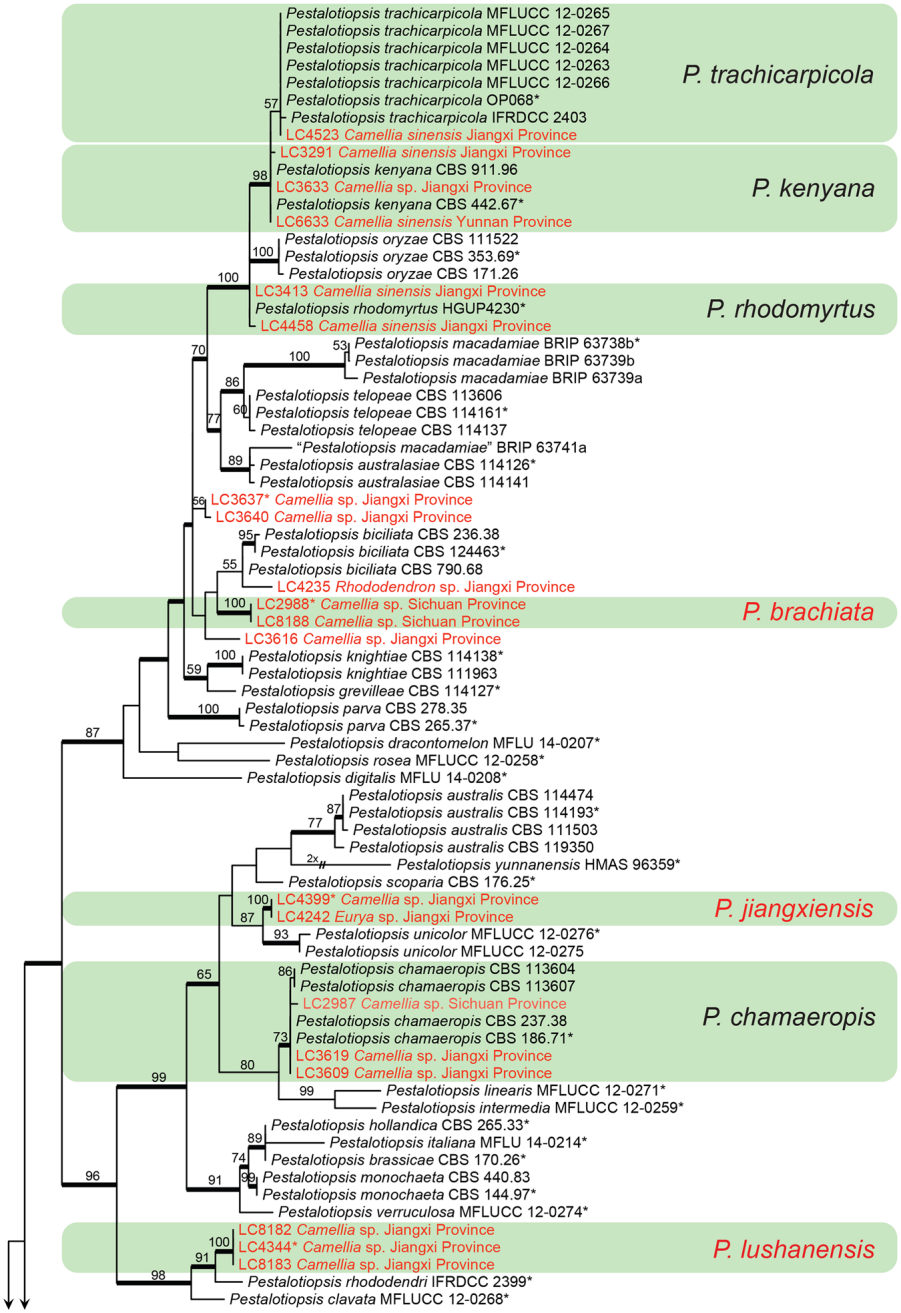
Part a of the phylogenetic tree of *Pestalotiopsis* calculated with maximum likelihood analysis on a combined dataset of three-locus sequences (ITS, TUB2, TEF) by running RAxML v.7.0.3, followed by Fig. 3. The RAxML bootstrap support values (>50%) are displayed at the nodes. Thickened branches indicate branches also present in the Bayesian tree with >0.95 posterior probabilities. Stars indicate ex-type cultures. Strains from *Camellia* spp. in this study are in red colour.

**Figure 3:**
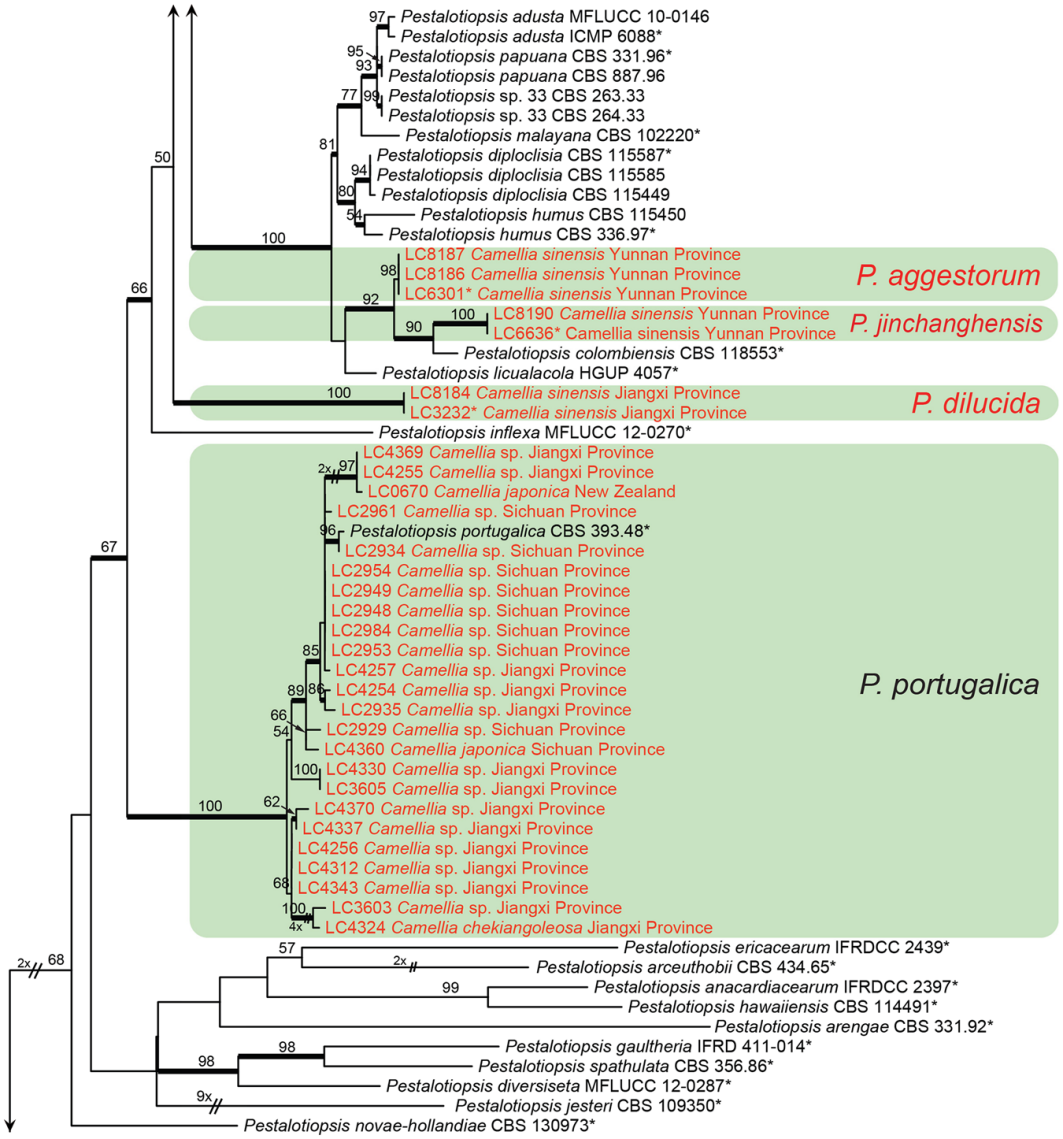
Part b of the phylogenetic tree of *Pestalotiopsis*, followed by Fig. 4.

**Figure 4:**
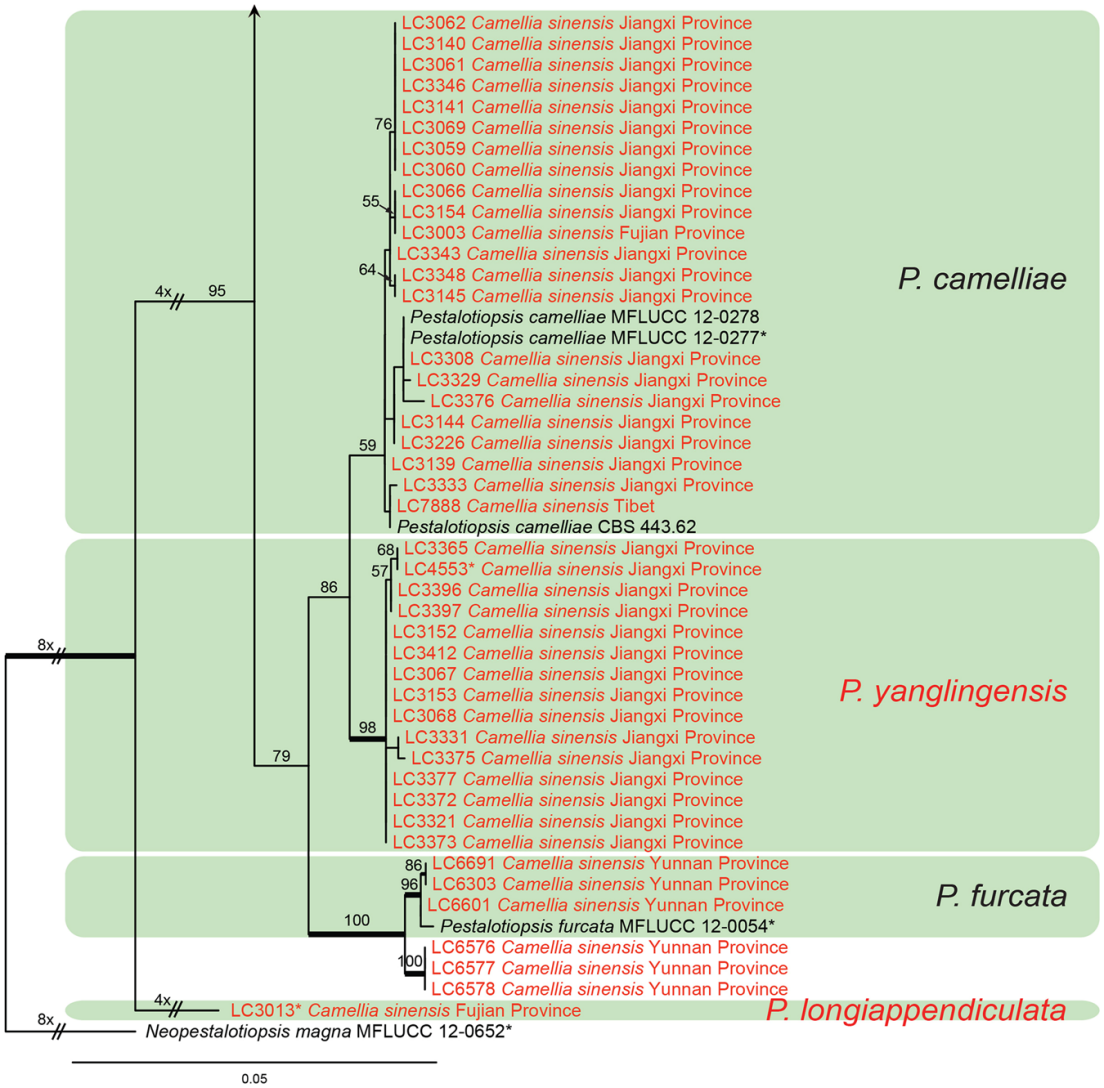
Part c of the phylogenetic tree of *Pestalotiopsis*.

Phylogenetic analyses of 180 isolates of *Pestalotiopsis* were performed on the single locus and concatenated dataset of ITS, TUB2 and TEF, with *Neopestalotiopsis magna* (MFLUCC 12-0652) as outgroup (Figs 2–4, Supplementary Figs S4–6). The concatenated dataset contained 494 characters with alignment gaps for ITS, 800 for TUB2, and 545 for TEF. For the Bayesian inference, a GTR+I+G model with invgamma-distributed rate was selected for ITS, HKY+I+G model with invgamma-distributed rate for TUB2 and TEF. The maximum likelihood tree confirmed the tree topology of the Bayesian consensus tree. Isolates from *C. sinensis* and other *Camellia* spp. in *Pestalotiopsis* clustered in 19 clades (Figs 2–4), which represented seven known species (*P. trachicarpicola*, *P. kenyana*, *P. rhodomyrtus*, *P. chamaeropis*, *P. portugalica*, *P. camelliae*, *P. furcata*), eight potential new species. Six strains (LC3616, LC3637, LC3640, LC6576, LC6577, LC6578), separated into three clades, are undetermined due to their sterility, or undistinguishable morphological characters from their most closely related species on the phylogenetic tree (Figs 2, 4).

#### *Pseudopestalotiopsis*

**Figure 5:**
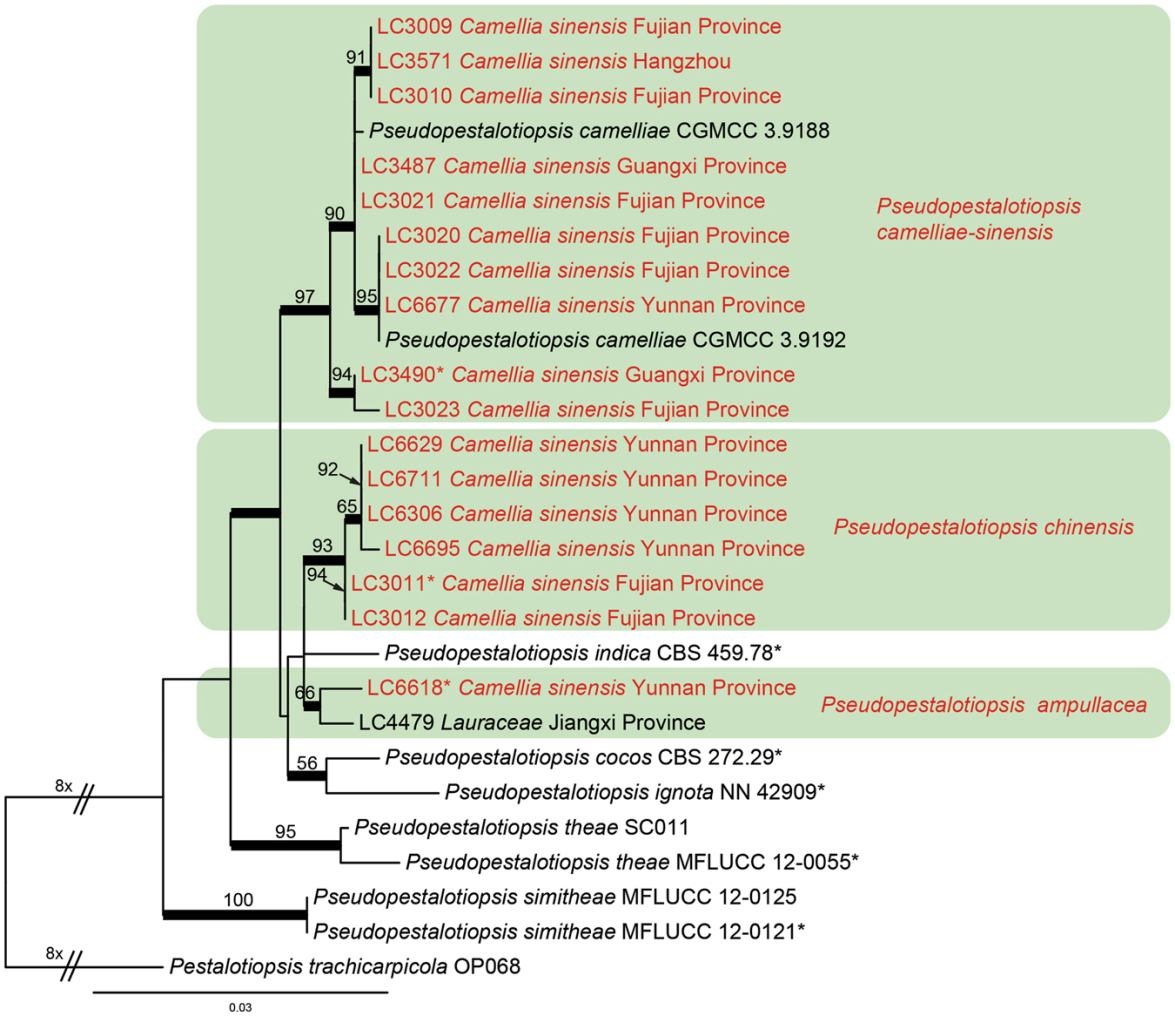
Phylogenetic tree of *Pseudopestalotiopsis* calculated with maximum likelihood analysis on a combined dataset of three-locus sequences (ITS, TUB2, TEF) by running RAxML v.7.0.3. The RAxML bootstrap support values (>50%) are displayed at the nodes. Thickened branches indicate branches also present in the Bayesian tree with >0.95 posterior probabilities. Stars indicate ex-type cultures. Strains from *Camellia* spp. in this study are in red colour.

Phylogenetic analyses of 27 isolates of *Neopestalotiopsis* were performed on single locus and concatenated datasets, with *Pestalotiopsis trachicarpicola* (OP068) as outgroup (Fig. 5, Supplementary Figs S7–9). The concatenated dataset contained 384 characters with alignment gaps for ITS, 479 for TEF, and 377 for TUB2. For the Bayesian inference, a GTR+G model with gamma-distributed rate was selected for ITS and TUB2, and HKY+G model with gamma-distributed rate for TEF. The maximum likelihood tree confirmed the tree topology of the Bayesian consensus tree. Isolates from *C. sinensis* in *Pseudopestalotiopsis* clustered in three clades.

### Taxonomy

**Figure 6:**
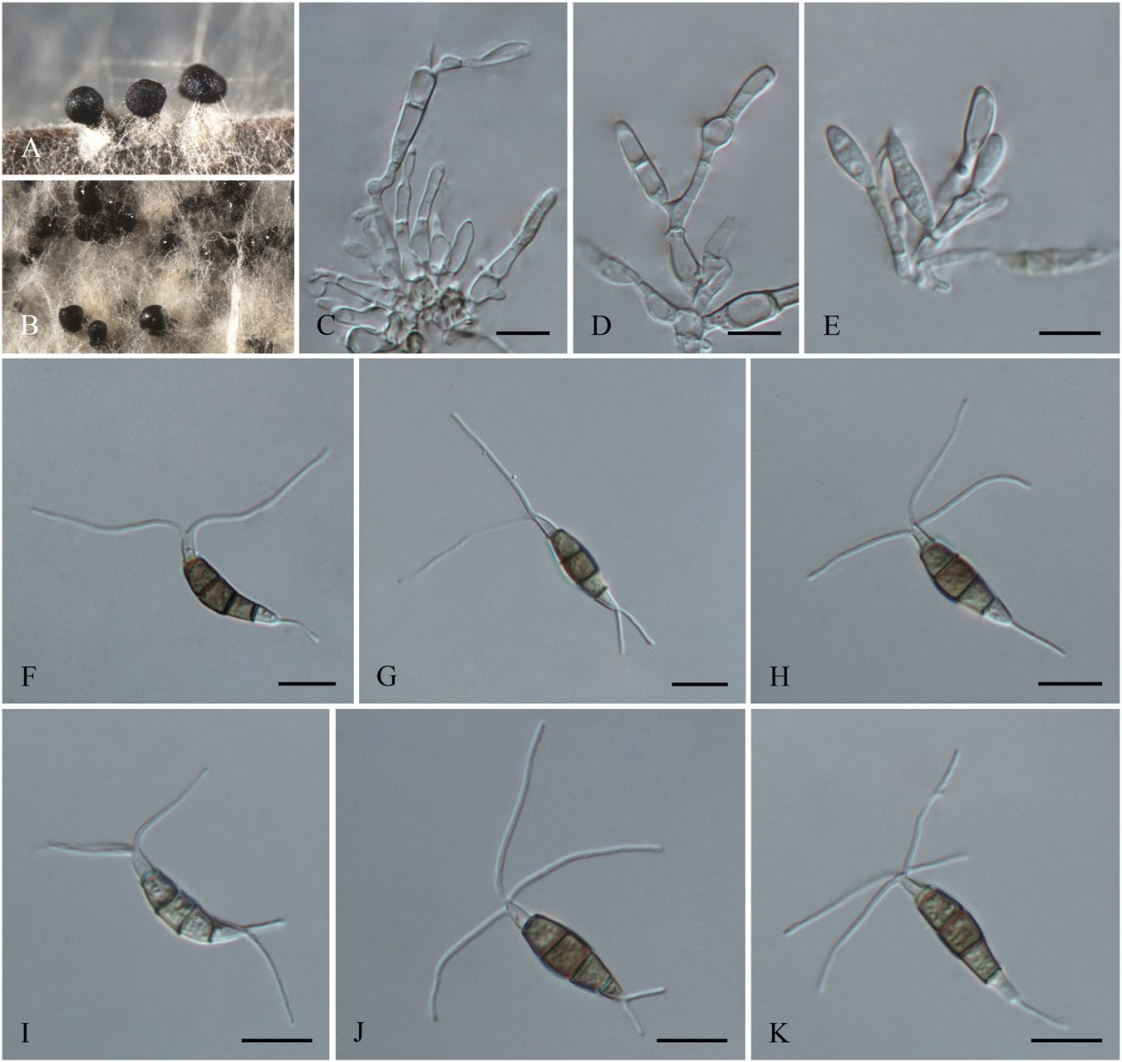
*Pestalotiopsis aggestorum* (from ex-holotype culture CGMCC 3.18159 (= LC6301)). (**A**) Conidiomata sporulating on pine needle. (**B**) Conidiomata sporulating on PDA. (**C**–**E**) Conidiogenous cells and conidia. (**F**–**K**) Conidia. Scale bars = 10 μm.

***Pestalotiopsis aggestorum*** F. Liu & L. Cai **sp. nov**. — MycoBank MB 818920 Fig. 6.

*Etymology*: refers to a landform, the terraces of a tea plantation.

Conidiomata pycnidial in culture on PDA, globose or clavate, aggregated or scattered, semi-immersed to erumpent, dark brown to black, 100–600 μm diam; exuding globose, dark brown to black conidial masses. Conidiophores reduced to conidiogenous cells; conidiogenous cells discrete or integrated, ampulliform, clavate or subcylindrical, hyaline, smooth, 6–14.7 × 2–5.5 μm. Conidia fusoid, ellipsoid, straight to slightly curved, 4-septate, 19–24.5 × 5–7 μm (av. ± SD = 21.5 ± 1.5 × 6.2 ± 0.6 μm); basal cell conic or obconic with a truncate base, hyaline, rugose and thin-walled, 2–5.5 μm long; three median cells doliiform, 11.5–16 μm (av. ± SD = 13.5 ± 0.9 μm) long, wall minutely verruculose, versicoloured, septa darker than the rest of cell (second cell from base pale brown, 3.5–5.5 μm long; third cell darker brown, 3.5–5.5 μm long; fourth cell brown, 4.5–6 μm long); apical cell 3.5–6.5 μm long, hyaline, obconic with a truncate base or subcylindrical, thin-walled, slightly rugose; with 2–3 tubular apical appendages (mostly 3), arising from the apical crest, unbranched (rarely branched), filiform, 18–28 μm; basal appendages 1–2, tubular, occasionally branched, centric, 5–14 μm long.

Culture characteristics: Colonies on PDA attaining72 mm diam after 7 d at 25 °C, with entire edge, whitish, with dense aerial mycelia on the surface with black, gregarious conidiomata; reverse whitish in colour.

Material examined: China, Yunnan Province, Xishuangbanna, Jingmai Mountain, on leaves of terraced tea *Camellia sinensis*, 16 Apr. 2015, *F. Liu*, *HMAS 247058* (holotype), **ex-holotype** living culture CGMCC 3.18159 (= LC6301); ibid. LC8186; ibid. LC8187.

Note: *Pestalotiopsis aggestorum* is phylogenetically most closely related to *P. colombiensis* and *P. jinchanghensis* (Fig. 3). However, the former species differs from *P. colombiensis* in the length of basal appendages (5–14 μm vs. 2–5 μm); differs from *P. jinchanghensis* in producing relatively shorter conidia (19–24.5 × 5–7 μm, av. = 21.5 μm vs. 21–30 × 5–7 μm, av. = 25.5 μm).

**Figure 7:**
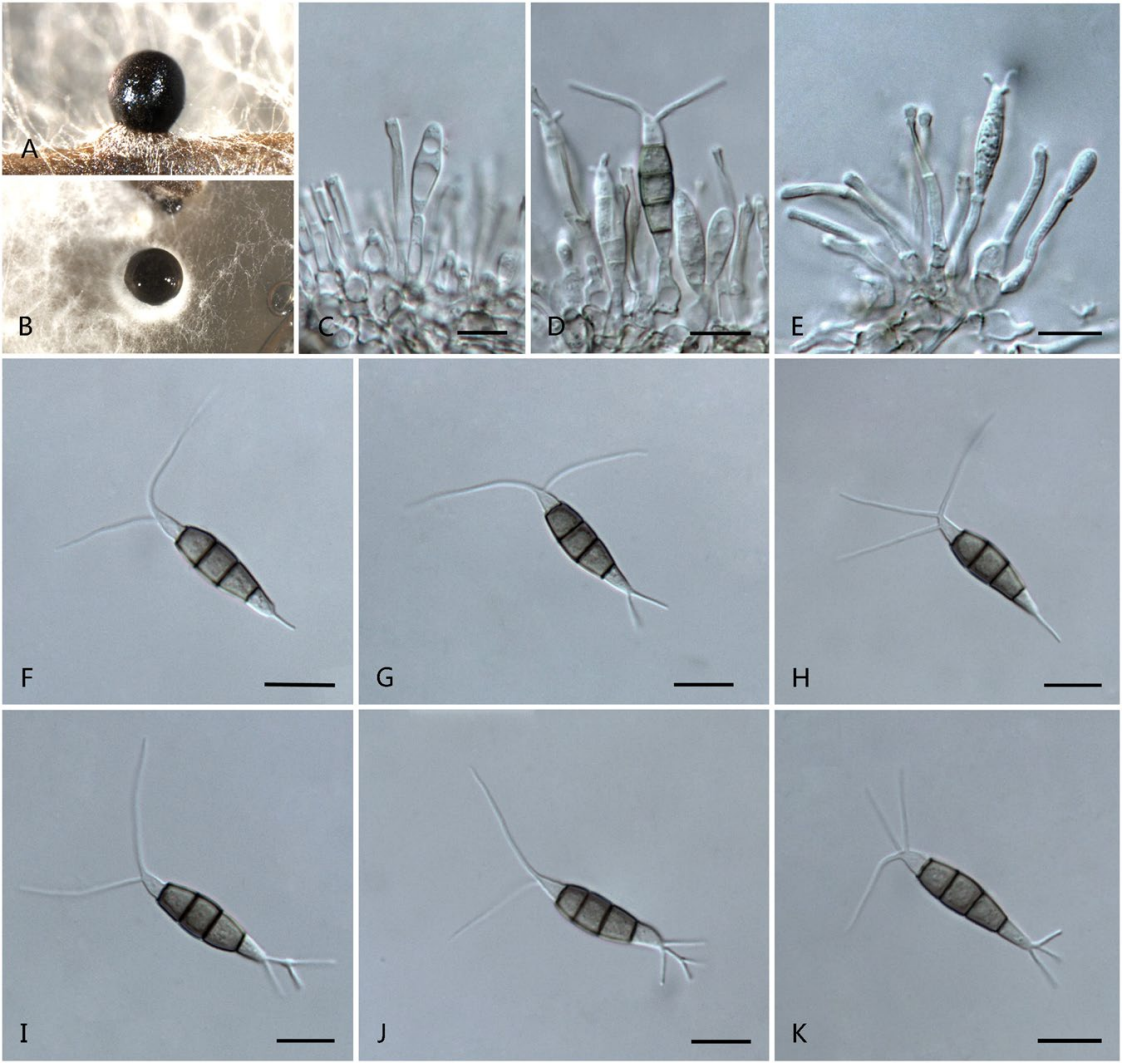
*Pestalotiopsis brachiata* (from ex-holotype culture CGMCC 3.18151 (= LC2988)). (**A**) Conidioma sporulating on pine needle. (**B**) Conidioma sporulating on PDA. (**C–E**) Conidiogenous cells and conidia. (**F–K**) Conidia. Scale bars = 10 μm.

***Pestalotiopsis brachiata*** F. Liu & L. Cai **sp. nov**. — MycoBank MB 818914 Fig. 7.

*Etymology*: refers to branched basal appendages.

Conidiomata pycnidial in culture on PDA, globose or clavate, aggregated or scattered, embedded or semi-immersed to erumpent, dark brown to black, up to 700 μm diam; exuding globose, dark brown to black conidial masses. Conidiophores sparsely septate at base, branched or unbranched, subcylindrical, hyaline, thin-walled, verruculose. Conidiogenous cell integrated, ampulliform, clavate or cylindrical, hyaline, smooth-walled, 15–30 × 2–8 μm. Conidia fusoid, ellipsoid to subcylindrical, straight to slightly curved, 4-septate, 23.5–25 × 6.5–8 μm (av. ± SD = 24.9 ± 1.4 × 7 ± 0.3 μm); basal cell conic to obconic with a truncate base, hyaline, rugose and thin-walled, 4–6.5 μm long; three median cells doliiform, 14–16.5 μm (av. ± SD = 15.5 ± 0.9 μm) long, wall minutely verruculose or rugose, concolourous, pale brown to brown, septa darker than the rest of cell (second cell from base 4.5–6 μm long; third cell 4.5–5.5 μm long; fourth cell 4–5.5 μm long); apical cell 3.5–6 μm long, hyaline, cylindrical, subcylindrical or obconic with a truncate base, thin-walled, slightly rugose; with 2–3 tubular apical appendages (mostly 2), arising from the apical crest, unbranched, filiform, 16–28.5 μm (av. ± SD = 22 ± 4.1 μm) long; basal appendage 1–4, tubular, branched or unbranched, centric, 5.5–9.5 μm long.

Culture characteristics: Colonies on PDA reaching 80 mm diam after 7 d at 25 °C, undulated at the edge, whitish to pale yellow-coloured, with dense aerial mycelia on surface, forming black, gregarious conidiomata; reverse whitish to pale yellow in color.

Material examined: China, Sichuan Province, Chengdu, on healthy leaves of *Camellia* sp., 5 Oct. 2012, *F. Liu*, *HMAS 247050* (holotype), **ex-holotype** culture CGMCC 3.18151 (= LC2988); ibid. LC8188.

Note: *Pestalotiopsis brachiata* is the only species producing more than two basal appendages (1–4, sometimes branched) in this genus, which notably distinguish it from all other species in *Pestalotiopsis*. Based on multi-locus tree, two strains of *P. brachiata* formed a well-supported clade, closely related to *P. biciliata* (Fig. 2). While two species differ by 5 bp differences in ITS and 15 bp in TEF.

***Pestalotiopsis camelliae*** Yan M. Zhang, Maharachch. & K.D. Hyde, Sydowia 64: 337. 2012.

Materials examined: China, Jiangxi Province, Chongyi County, Yangling National Forest Park, on leaves of *Camellia sinensis*, 24 Apr. 2013, *F. Liu*, living culture LC3059; ibid., LC3060; ibid., LC3061; ibid., LC3062; ibid., LC3066; ibid., LC3069; ibid., LC3139; ibid., LC3140; ibid., LC3141; ibid., LC3144; ibid., LC3145; ibid., LC3154; ibid., LC3226; ibid., LC3308; ibid., LC3329; ibid., LC3333; ibid., LC3343; ibid., LC3346; ibid., LC3348; ibid., LC3376; Tibet, Lulang town, on leaves of *C. sinensis*, 15 Jun. 2015, *F. Liu*, living culture LC7888; Fujian Province, Zhangzhou, on leaves of *C. sinensis*, Nov. 2012, *F. Liu*, living culture LC3003.

Notes: *Pestalotiopsis camelliae* is currently only known from *Camellia* ^5, 18^.

***Pestalotiopsis chamaeropis*** Maharachch., K.D. Hyde & Crous, Stud. Mycol. 79: 158. 2014.

Materials examined: China, Sichuan Province, Chengdu Botanical Garden, on healthy leaves of *Camellia* sp., 5 Oct. 2012, *F. Liu*, LC2987; Jiangxi Province, Gannan Normal University, on *Camellia* sp., *N. Zhou*, 7 Sep. 2013, LC3619; Jiujiang, Lushan Botanical Garden, 3 sep. 2013, *N. Zhou*, LC3609.

**Figure 8:**
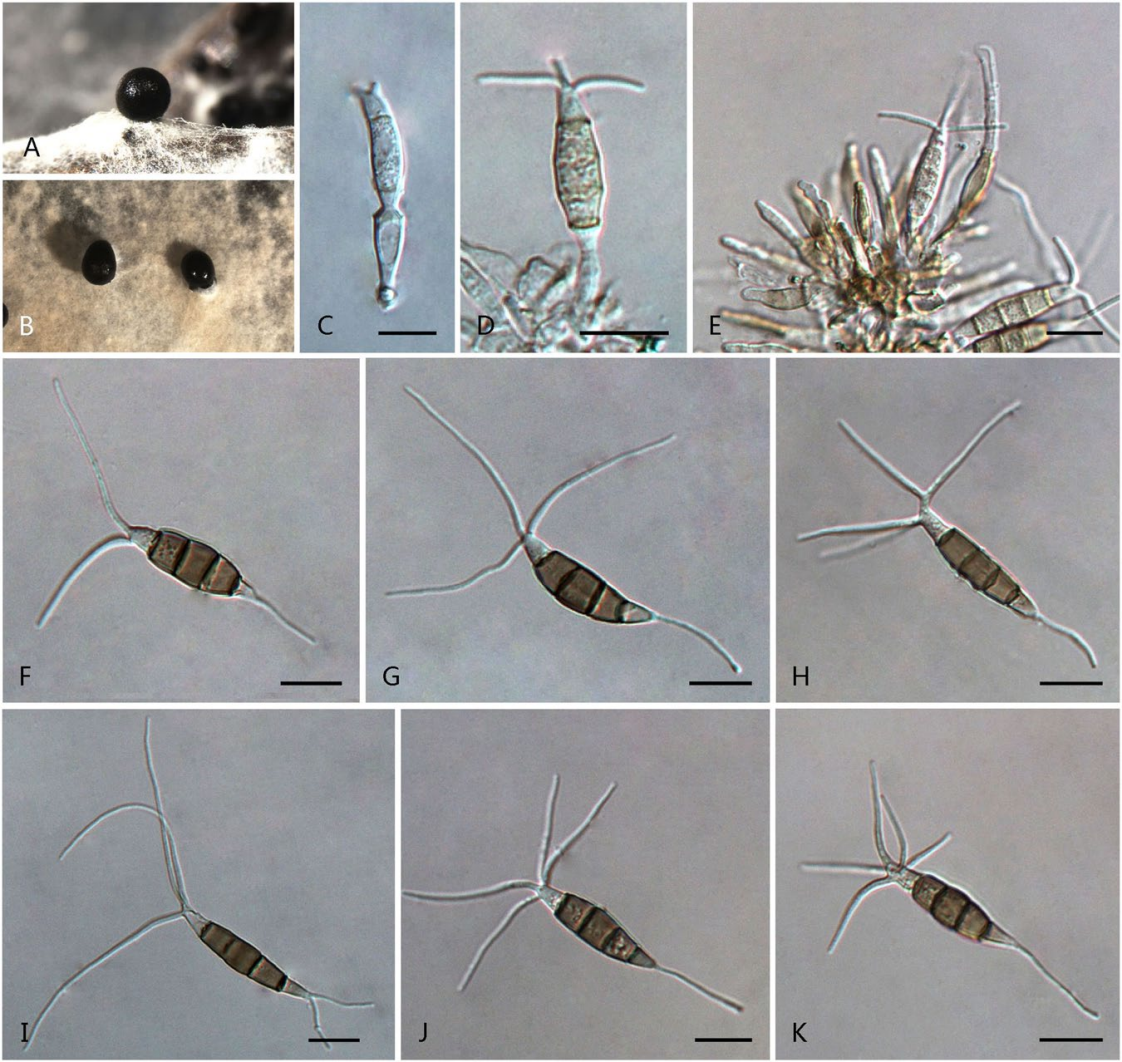
*Pestalotiopsis dilucida* (from ex-holotype culture CGMCC 3.18154 (= LC3232)). (**A**) Conidioma sporulating on pine needle. (**B)** Conidiomata on PDA. (**C–E**) Conidiogenous cells and conidia. (**F–K**) Conidia. Scale bars = 10 μm.

***Pestalotiopsis dilucida*** F. Liu & L. Cai **sp. nov**. — MycoBank MB 818915 Fig. 8.

*Etymology*: *dilucida*, named after the distinctly phylogenetic status.

Conidiomata pycnidial in culture on PDA, globose or clavate, scattered, semi-immersed to erumpent, dark brown to black, up to 750 μm diam; exuding globose, dark brown to black conidial masses. Conidiophores often reduced to conidiogenous cells, sparsely septate at the base and branched or unbranched. Conidiogenous cell mostly integrated, ampulliform, clavate or cylindrical, hyaline, smooth-walled, 7.5–20 × 2–4 μm. Conidia fusoid, ellipsoid, straight to slightly curved, 4-septate, 24.5–32 × 5.5–8.5 μm (av. ± SD = 27.3 ± 2.27 × 6.76 ± 0.91 μm); basal cell conic to obconic with a truncate base, hyaline, rugose and thin-walled, 5–7 μm long; three median cells doliiform, 15–19.5 μm (av. ± SD = 17.2 ± 1.45 μm) long, wall smoothly, minutely verruculose, concolourous, pale brown to brown, septa darker than the rest of cell (second cell from base 4.5–6.5 μm long; third cell 4.5–6.5 μm long; fourth cell 4.5–7 μm long); apical cell 3.5–5.5 μm long, hyaline, cylindrical, subcylindrical or obconic with a truncate base, thin-walled, slightly rugose; with 2–5 tubular apical appendages (mostly 3), arising from the apical crest, unbranched or branched, filiform, (10–)16.5–39.5 μm (av. ± SD = 23.1 ± 5.6 μm) long; basal appendage 1–2, tubular, unbranched, centric, 5.5–20(–28.5) μm long.

Culture characteristics: Colonies on PDA reaching 70 mm diam after 7 d at 25 °C, with undulated at the edge, whitish, with dense aerial mycelia on surface, forming black, gregarious conidiomata; reverse whitish.

Material examined: China, Jiangxi Province, Chongyi County, Yangling National Forest Park, on leaves of *Camellia sinensis*, 24 Apr. 2013, *F. Liu*, *HMAS 247053* (holotype), **ex-holotype** culture CGMCC 3.18154 (= LC3232); ibid. LC8184.

Notes: *Pestalotiopsis dilucida* is apparently a distinct species (Fig. 3). Sequences of the ex-type of *P. dilucida* show 98% identity in ITS, 95% in TUB2, and 95% in TEF to its most closely related species *P. licualacola*.

***Pestalotiopsis furcate*** Maharachch. & K.D. Hyde, Mycotaxon 123: 54. 2013.

Materials examined: China, Yunnan Province, Gua Feng Zhai, on *Camellia sinensis*, 18 Apr. 2015, *F. Liu*, LC6601 = LF1245; Tian Ba, on healthy leaves of *C. sinensis*, 21 Apr. 2015, *F. Liu*, LC6303 = LF1310; Gaoshan, Huang Zhu Peng, on *C. sinensis*, 21 Apr. 2015, *F. Liu*, LC6691 = LF1371.

**Figure 9:**
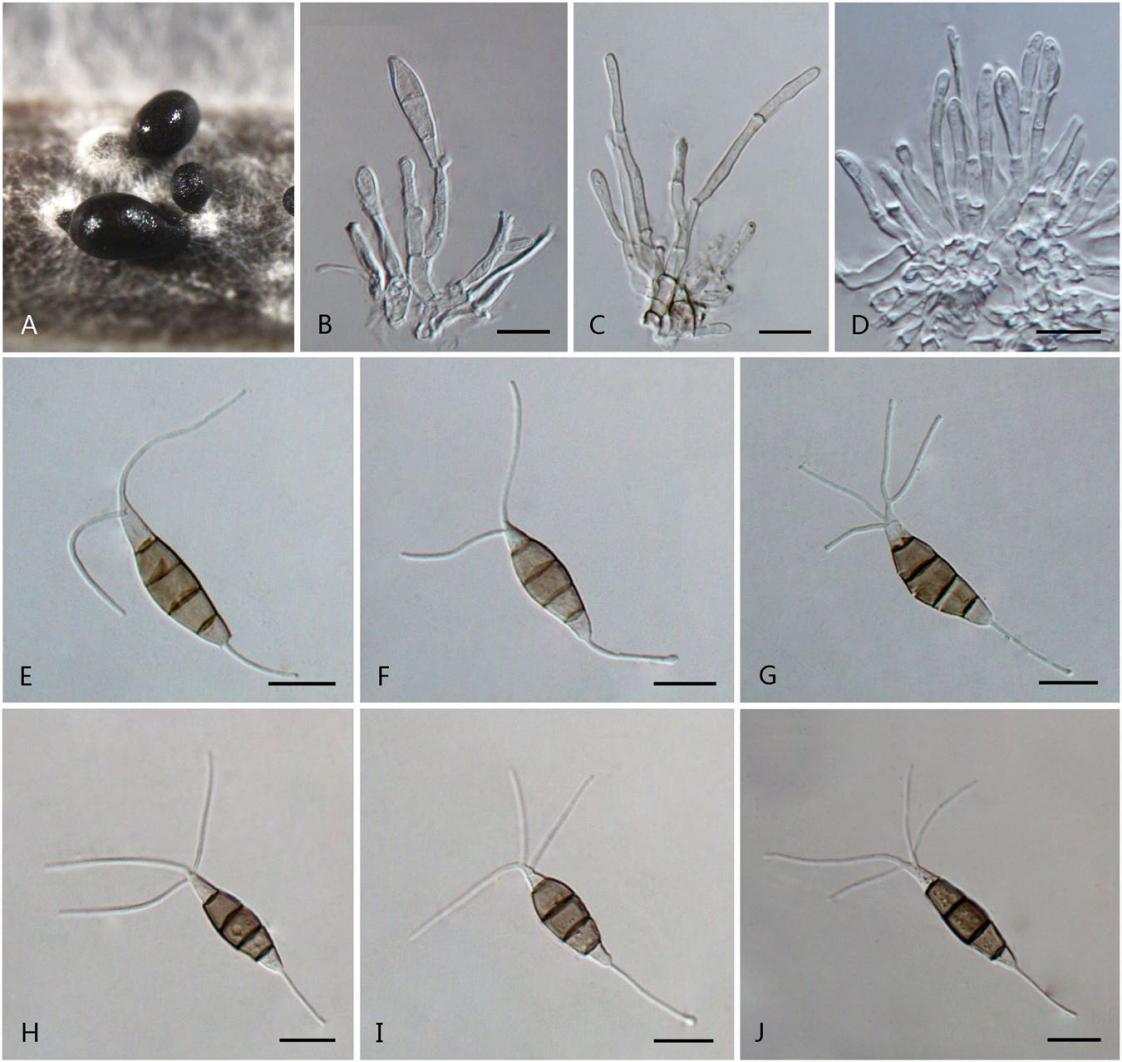
*Pestalotiopsis jiangxiensis* (from ex-holotype culture CGMCC 3.18218 (= LC4399)). (**A**) Conidiomata sporulating on pine needle. (**B–D**) Conidiophores. (**E–J**) Conidia. Scale bars = 10 μm.

***Pestalotiopsis jiangxiensis*** F. Liu & L. Cai **sp. nov**. — MycoBank MB 818916 Fig. 9.

*Etymology*: named after the country where it was collected from, Jiangxi Province.

Conidiomata pycnidial on pine needle, globose or clavate, aggregated or scattered, embedded or semi-immersed to erumpent, dark brown to black; exuding globose, dark brown to black conidial masses; on PDA conidiomata usually covered under aerial mycelia. Conidiophores branched or unbranched, hyaline or light brown, thin-walled, verruculose, up to 55 μm long. Conidiogenous cell integrated, ampulliform, clavate, obclavate or cylindrical, hyaline or rarely light brown, smooth-walled, 11.5–29.5 × 2–5.5 μm. Conidia fusoid to broadly fusoid, subcylindrical, straight to slightly curved, 4-septate, 22–29 × 6–9 μm (av. ± SD = 25.7 ± 1.7 × 7.3 ± 0.7 μm); basal cell conic to obconic with a truncate base, hyaline or light brown, rugose and thin-walled, 3.5–6.5 μm long; three median cells doliiform, 12.5–19 μm (av. ± SD = 15.9 ± 1.5 μm) long, wall minutely verruculose or rugose, concolourous, pale brown to brown, septa darker than the rest of cell (second cell from base 3.5–6 μm long; third cell 4.5–6.5 μm long; fourth cell 4–7 μm long); apical cell 3.5–7 μm long, hyaline, cylindrical, subcylindrical or obconic with a truncate base, thin-walled, slightly rugose; with 2–4 tubular apical appendages (mostly 3), arising from the apical crest, but inserted at different locus in the upper half of the apical cell, rarely branched, filiform, 16.5–32 μm (av. ± SD = 22.4 ± 3.8 μm) long; single basal appendage, tubular, unbranched, centric, 6.5–19.5 μm long.

Culture characteristics: Colonies on PDA reaching 80 mm diam after 7 d at 25 °C, undulated at the edge, whitish to pale yellow, with dense aerial mycelia on surface, forming black, gregarious conidiomata under aerial mycelia; reverse whitish to pale yellow.

Materials examined: China, Jiangxi Province, Lushan National Park, on *Camellia* sp., 5 Sep. 2013, *Y.H. Gao*, *HMAS 247060* (holotype), **ex-holotype** culture CGMCC 3.18218 (= LC4399); on *Eurya* sp., 5 Sep. 2013, *Y.H. Gao*, LC4242.

Notes: *Pestalotiopsis jiangxiensis* is phylogenetically most closely related to *P. unicolor* (Fig. 2). The former differs in producing broadly fusoid conidia that are longer and wider (22–29 × 6–9 μm, av. = 25.7 × 7.3 μm vs. 20–24.5 × 4–6 μm, av. = 22 × 5.1 μm). Moreover, the apical appendages of *P. jiangxiensis* are from different loci in the upper half of the apical cell, but that of *P. unicolor* arise from the apex of apical cell^16^.

**Figure 10:**
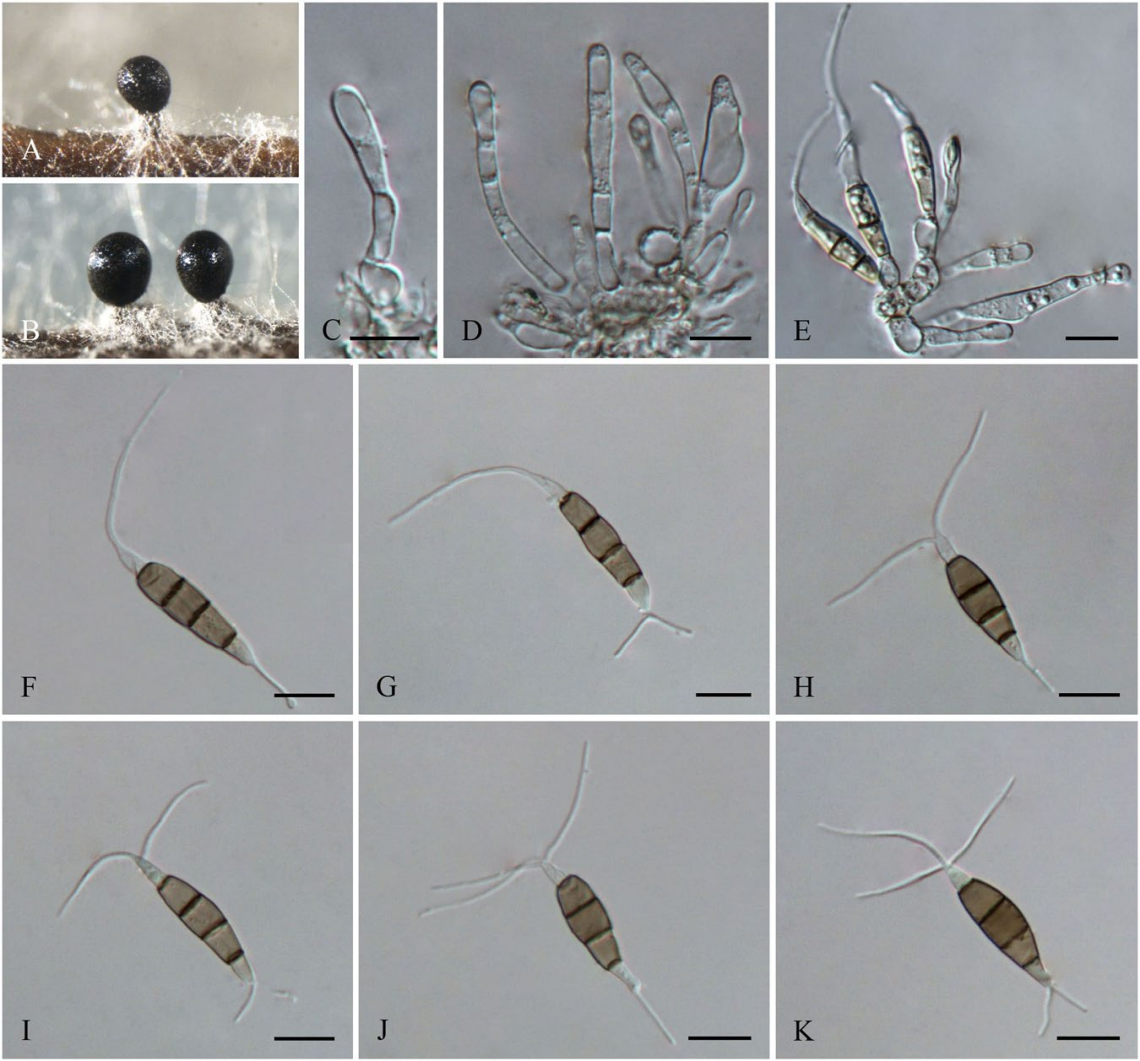
*Pestalotiopsis jinchanghensis* (from ex-holotype culture CGMCC 3.18158 (= LC6636)). (**A**) Conidioma sporulating on pine needle. (**B**) Conidiomata sporulating on PDA. (**C–E**) Conidiogenous cells and conidia. (**F–K**) Conidia. Scale bars = 10 μm.

***Pestalotiopsis jinchanghensis*** F. Liu & L. Cai **sp. nov**. — MycoBank MB 818917 Fig. 10.

*Etymology*: named after the location where it was collected from, Jinchanghe, Yunnan Province.

Conidiomata pycnidial in culture on PDA, globose or clavate, scattered, semi-immersed to erumpent, dark brown to black, up to 650 μm diam; exuding globose, dark brown to black conidial masses. Conidiophores often reduced to conidiogenous cell, occasionally septate and branched at the base. Conidiogenous cells mostly integrated, spheriform, cylindrical, or clavate, hyaline, smooth-walled, 5–12 × 2–7 μm. Conidia fusoid, ellipsoid, straight, 4-septate, 22–32 × 5.5–8.5 μm (av. ± SD = 26.5 ± 2.5 × 6.5 ± 0.65 μm); basal cell subcylindrical, conic to obconic with a truncate base, hyaline, rugose and thin-walled, 3–6 μm long; three median cells doliiform or cylindrical, 15–20.5 μm (av. ± SD = 17 ± 1.7 μm) long, smooth-walled, minutely verruculose, concolourous, brown to olivaceous, septa darker than the rest of cell (second cell from base 4–7.5 μm long; third cell 4–7.5 μm long; fourth cell 5–7.5 μm long); apical cell 3.5–7 μm long, hyaline, subcylindrical or obconic with a truncate base, thin-walled, slightly rugose; with 1–3 tubular apical appendages (mostly 2), arising from the apical crest, unbranched, filiform, 15–33.5 μm (av. ± SD = 21.5 ± 4.2 μm) long; basal appendage 1–2, tubular, unbranched, centric, 5.5–15.5 μm long.

Culture characteristics: Colonies on PDA reaching 65 mm diam after 7 d at 25 °C, with smooth edge, whitish to pale honey-coloured, with dense aerial mycelia on surface, forming black, gregarious conidiomata; reverse whitish to pale honey.

Material examined: China, Yunnan Province, Xishuangbanna, Jinchanghe, on leaves of *Camellia sinensis*, 20 Apr. 2015, *F. Liu*, *HMAS 247061* (holotype), **ex-holotype** living culture CGMCC 3.18158 (= LC6636); ibid. LC8190.

Note: *Pestalotiopsis jinchanghensis* is phylogenetically most closely related to *P. colombiensis* (Fig. 3), but differs in producing longer basal appendages (5.5–15.5 μm vs. 2–5 μm).

***Pestalotiopsis kenyana*** Maharachch., K.D. Hyde & Crous, Stud. Mycol. 79: 166. 2014.

Materials examined: China, Yunnan Province, Jin Chang He, on *Camellia sinensis*, 20 Apr. 2015, *F. Liu*, LC6633; Jiangxi Province, Gannan Normal University, on *Camellia* sp., 7 Sep. 2013, *N. Zhou*, LC3633; Chongyi County, Yangling National Forest Park, on *C. sinensis*, 24 Apr. 2013, *F. Liu*, LC3291.

**Figure 11:**
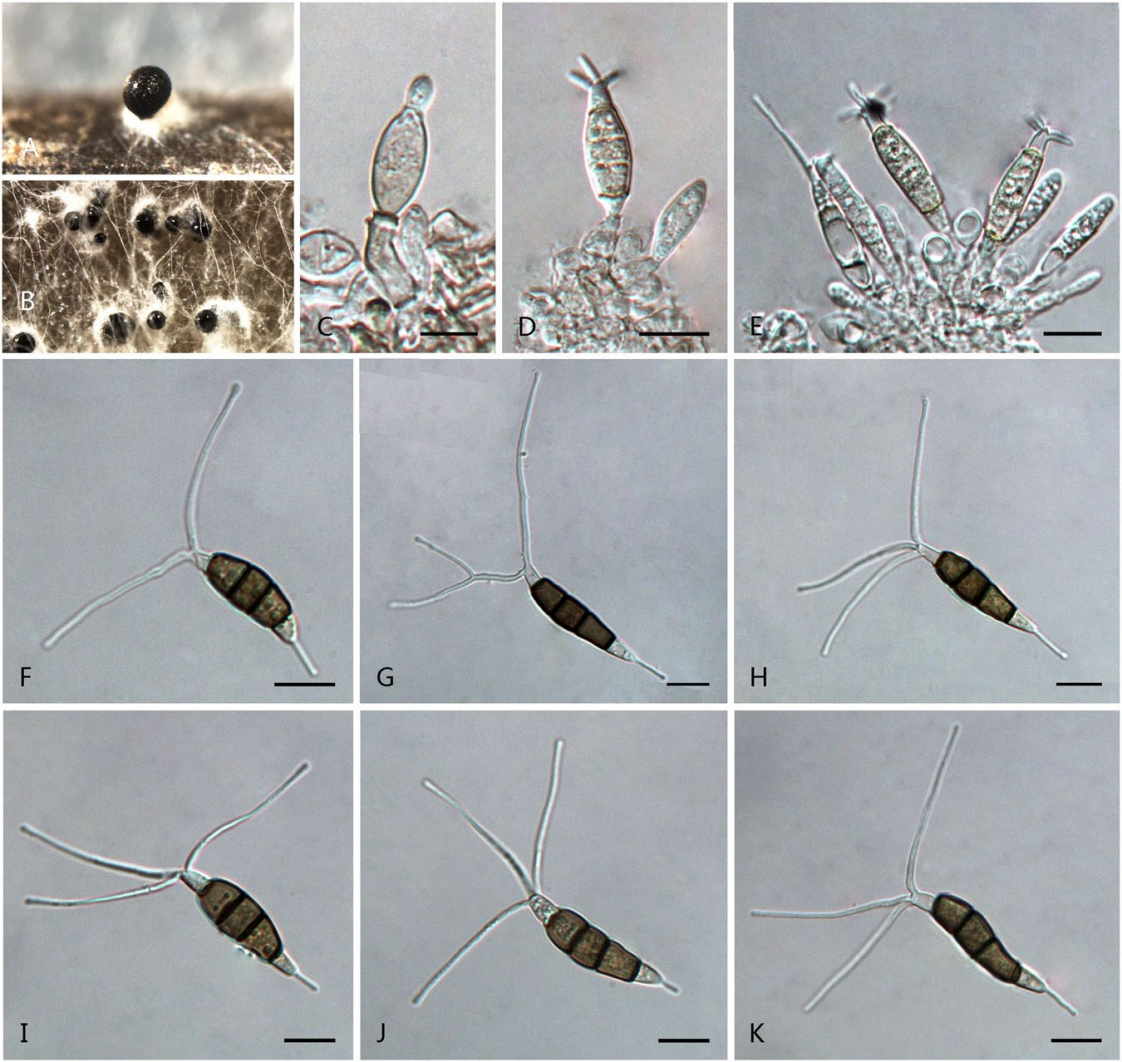
*Pestalotiopsis longiappendiculata* (from ex-holotype culture CGMCC 3.18153 (= LC3013)). (**A**) Conidioma sporulating on pine needle. (**B**) Conidiomata sporulating on PDA. (**C–E**) Conidiogenous cells and conidia. (**F–K**) Conidia. Scale bars = 10 μm.

***Pestalotiopsis longiappendiculata*** F. Liu & L. Cai **sp. nov**. — MycoBank MB 818918 Fig. 11.

Etymology: refers to the long apical appendages of this species.

Conidiomata pycnidial in culture on PDA, globose or clavate, aggregated or scattered, embedded or semi-immersed to erumpent, dark brown to black, 50–450 μm diam; exuding globose, dark brown to black conidial masses. Conidiophores sparsely septate at base, branched or unbranched, subcylindrical, hyaline, smooth, up to 20 μm. Conidiogenous cell integrated, ampulliform or cylindrical, hyaline, smooth-walled, 20–34 × 3.5–5.5 μm. Conidia fusoid, ellipsoid, straight to slightly curved, 4-septate, 28–34 × 7–10 μm (av. ± SD = 31.8 ± 2.0 × 8.3 ± 0.8 μm); basal cell conic to obconic with a truncate base, hyaline, rugose and thin-walled, 3.5–8 μm long; three median cells doliiform to subcylindrical, 18–22 μm (av. ± SD = 20.6 ± 1.3 μm) long, wall minutely rugose, concolourous, pale brown to brown, septa darker than the rest of cell (second cell from base 6.5–9 μm long; third cell 5–7.5 μm long; fourth cell 5.5–7.5 μm long); apical cell 4–7 μm long, hyaline, cylindrical, subcylindrical or obconic with a truncate base, thin-walled, slightly rugose; with 2–3 tubular apical appendages, arising from the apical crest, branched or unbranched, filiform, 29.5–47.5 μm (av. ± SD = 37.5 ± 4.6 μm) long; single basal appendage, tubular, unbranched, centric, 3.5–9 μm long.

Culture characteristics: Colonies on PDA reaching 80 mm diam after 7 d at 25 °C, undulated at the edge, whitish to pale yellow-coloured, with dense aerial mycelia on surface, forming black, gregarious conidiomata; reverse whitish to pale yellow.

Material examined: China, Fujian Province, Zhangzhou, on *Camellia sinensis*, Nov. 2012, *L. Cai*, *HMAS 247052* (holotype), **ex-holotype** living culture CGMCC 3.18153 (= LC3013).

Notes: *Pestalotiopsis longiappendiculata* is morphologically comparable to *P. brassicae* and *P. novae-hollandice* in respect to their long apical appendages. *P. longiappendiculata* however, differs from the latter two in producing less appendages (2–3 vs. 3–5 in *P. brassicae* and 3–9 in *P. novae-hollandiae*), and further from *P. brassicae* in the shorter basal appendages (3.5–9 μm vs. 10–25 μm) and branched apical appendages^18^.

**Figure 12:**
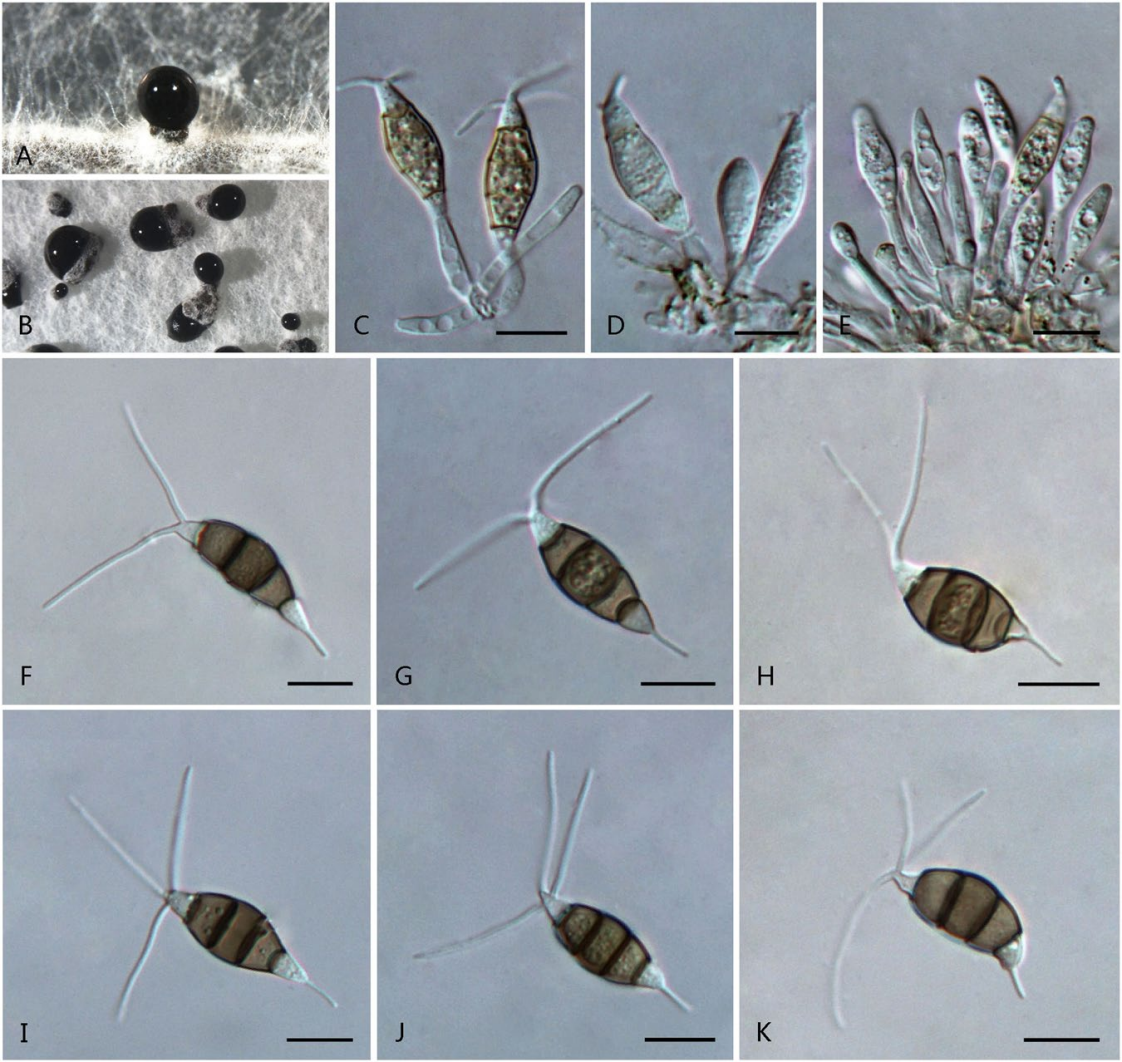
*Pestalotiopsis lushanensis* (from ex-holotype culture CGMCC 3.18160 (= LC4344)). (**A**) Conidioma sporulating on pine needle. (**B**) Conidiomata sporulating on PDA. (**C–E**) Conidiogenous cells and conidia. (**F–K**) Conidia. Scale bars = 10 μm.

***Pestalotiopsis lushanensis*** F. Liu & L. Cai **sp. nov**. — MycoBank MB 818919 Fig. 12.

*Etymology*: named after the location where it was collected from, Lushan, Jiangxi Province.

Conidiomata pycnidial in culture on PDA, globose or clavate, aggregated or scattered, semi-immersed to erumpent, dark brown to black, up to 750 μm diam; exuding globose, dark brown to black conidial masses. Conidiophores reduced to conidiogenous cell; when present, septate near base, unbranches or branched at bases, subcylindrical, hyaline. Conidiogenous cell discrete or integrated, ampulliform, clavate or subcylindrical, hyaline, smooth-walled, wide at base, 8–40 × 4–6 μm. Conidia fusoid, ellipsoid, straight to slightly curved, 4-septate, slightly constricted at septa, 20–27 × 7.5–10 μm (av. ± SD = 22.3 ± 1.9 × 8.6 ± 0.6 μm); basal cell conic to obconic with a truncate base, hyaline, verruculose and thin-walled, 3–6 μm long; three median cells doliiform, 12–16.5 μm (av. ± SD = 14.2 ± 1.1 μm long), wall minutely verruculose, concolourous, pale brown to brown, septa darker than the rest of cell (second cell from base 3–5 μm long; third cell 3.5–6.5 μm long; fourth cell 3.5–6 μm long); apical cell 3.5–5.5 μm long, hyaline, obconic with a truncate base, thin-walled, verruculose; with 2–3 tubular apical appendages (mostly 3), arising from the apical crest, unbranched, filiform, 17–26 μm (av. ± SD = 20.3 ± 2.9 μm) long; basal appendage single, tubular, unbranched, centric, 4–6.5 μm long.

Culture characteristics: Colonies on PDA attaining 60 mm diam after 7 d at 25 °C, with undulate edge, pale honey-coloured, sparse aerial mycelia on the surface with black, gregarious conidiomata; reverse pale honey.

Material examined: China, Jiangxi Province, Lushan National Park, on *Camellia* sp., 5 Sep. 2013, *Y.H. Gao*, *HMAS 247059* (holotype), **ex-holotype** living culture CGMCC 3.18160 (= LC4344); ibid. LC8182; ibid. LC8183.

Notes: *Pestalotiopsis lushanensis* is most closely related to *P. rhododendri* and *P. clavata* (Fig. 2), but differs from *P. rhododendri* in forming longer apical (17–26 μm vs. 7.5–14.9 μm) and basal (4–6.5 μm vs. 2.8–4.9 μm) appendages, and from *P. clavata* in the shorter basal appendages (4–6.5 μm vs. 7–9 μm).

***Pestalotiopsis portugalica*** Maharachch., K.D. Hyde & Crous, Stud. Mycol. 79: 176. 2014.

Materials examined: China, Jiangxi Province, Lushan National Park, on *Camellia* sp., 5 Sep. 2013, *Y.H. Gao*, LC4254; ibid. LC4255; ibid. LC4256; ibid. LC4257; ibid. LC4312; ibid. LC4330; ibid. LC4337; ibid. LC4343; ibid. LC4369; ibid. LC4370; ibid. LC3603; ibid. LC3605; Lushan National Park, on *C. chekiangoleosa*, 5 Sep. 2013, *Y.H. Gao*, LC4324; Lushan National Park, on *C. japonica*, 5 Sep. 2013, *Y.H. Gao*, LC4360; Sichuan Province, Chengdu Botanical Garden, on *Camellia* sp., 5 Oct. 2012, *F. Liu*, LC2929; ibid. LC2934; ibid. LC2935; ibid. LC2949; ibid. LC2948; ibid. LC2953; ibid. LC2954; ibid. LC2961; Chengdu Botanical Garden, on healthy lives of *Camellia* sp., 5 Oct. 2012, *F. Liu*, LC2984. New Zealand, Mt Albert, on *C. japonica*, *C.F. Hill*, LC0670 (= ICMP 13999).

Notes: Although there are a few small subclades in *P. portugalica* cluster on the multi-locus tree (Fig. 3), we did not find solid evidence to support their taxonomic separation. *Pestalotiopsis portugalica* is currently only known from *Camellia*.

***Pestalotiopsis rhodomyrtus*** Y. Song, K. Geng, K.D. Hyde & Yong Wang, Phytotaxa 126: 27. 2013.

Materials examined: China, Jiangxi Province, Nanchang, Mei Ling, on *Camellia sinensis*, 24 Apr. 2013, *F. Liu*, LC3413; Yangling National Forest Park, on *C. sinensis*, 5. Sep. 2013, *Y.H. Gao*, LC4458.

***Pestalotiopsis trachicarpicola*** Y.M. Zhang & K.D. Hyde, Cryptog. Mycol. 33: 315. 2012.

Material examined: China, Jiangxi Province, Yangling National Froest Park, on *Camellia sinensis*, 5 Sep. 2013, *F. Liu*, LC4523.

**Figure 13:**
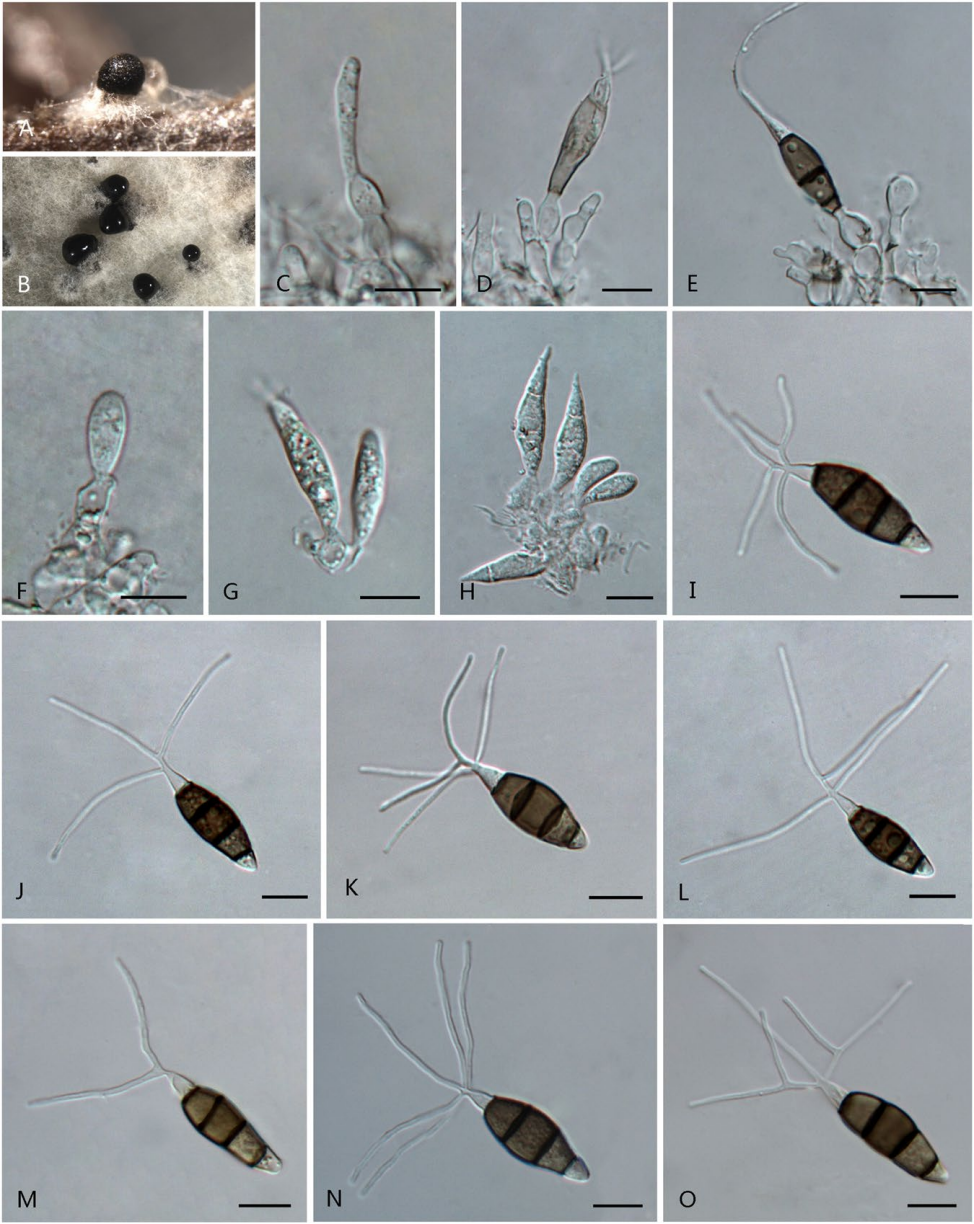
*Pestalotiopsis yanglingensis* (**A–E**, **M**–**O**) from ex-holotype culture CGMCC 3.18219 (= LC4553), (**F**–**L**) from culture LC3372. (**A**) Conidioma sporulating on pine needle. (**B**). Conidiomata sporulating on PDA. (**C–H**) Conidiogenous cells and conidia. (**I–O**) Conidia. Scale bars = 10 μm.

***Pestalotiopsis yanglingensis*** F. Liu & L. Cai **sp. nov**. — MycoBank MB 818921 Fig. 13.

*Etymology*: named after Yangling National Forest Park, where the holotype was collected.

Conidiomata pycnidial in culture on PDA, globose or clavate, aggregated or solitary, embedded, semi-immersed to erumpent, dark brown to black, 100–450 μm diam; exuding globose, dark brown to black conidial masses. Conidiophores often reduced to conidiogenous cell; when present, 1–3 septates, unbranched or irregularly branched at the base, subcylindrical, hyaline, up to 35 μm long. Conidiogenous cell discrete or integrated, obpyriform, ampulliform, cylindrical or subcylindrical, hyaline, smooth-walled, 11.5–36 × 3–8 μm. Conidia fusoid, ellipsoid, straight to slightly curved, 4-septate, slightly constricted at septa, 25–36 × 7–11 μm (av. ± SD = 29.4 ± 1.8 × 8.9 ± 0.78 μm); basal cell conic to obconic with a truncate base, hyaline, verruculose and thin-walled, 3–6 μm long; three median cells doliiform, 18–26 μm (av. ± SD = 20.5 ± 1.4 μm) long, wall minutely verruculose, concolourous, pale brown to brown, septa darker than the rest of cell (second cell from base 5–7 μm long; third cell 6–8 μm long; fourth cell 6–7.5 μm long); apical cell 3.5–7 μm long, hyaline, obconic with a truncate base, thin-walled, verruculose; with 2–7 tubular apical appendages (mostly 4), arising from the apex of the apical cell or inserted at different loci in the upper half of the apical cell, unbranched or branched, filiform, 16–30 μm (av. ± SD = 25.3 ± 2.92 μm) long; lacking basal appendage.

Culture characteristics: Colonies on PDA attaining 70 mm diam after 7 d at 25 °C, with lobate edge, whitish to pale honey-coloured, with sparse aerial mycelia on the surface with black conidiomata; reverse whitish to pale honey.

Materials examined: China, Jiangxi Province, Chongyi county, Yangling National Froest Park, on *Camellia sinensis*, 5 Sep. 2013, *Y.H. Gao*, *HMAS 247057* (holotype), **ex-holotype** living culture CGMCC 3.18219 (= LC4553); on *C. sinensis*, 24 Apr. 2013, *F. Liu*, living culture LC3067; ibid. LC3068; ibid. LC3152; ibid. LC3153; ibid. LC3321; ibid. LC3331; ibid. LC3365; ibid. LC3372; ibid. LC3373; ibid. LC3375; ibid. LC3377; ibid. LC3396; ibid. LC3397; ibid. LC3412.

Notes: *Pestalotiopsis yanglingensis* is a distinct species forming a separate clade in a sister position to *P. camelliae* (Fig. 4), and they could be distinguished from each other at 13 nucleotide sites among the TUB2 and TEF sequences. The former species is morphologically distinct from *P. camelliae* in producing higher ratio of branched apical appendages (rarely branched in *P. camelliae*)^5^.

**Figure 14:**
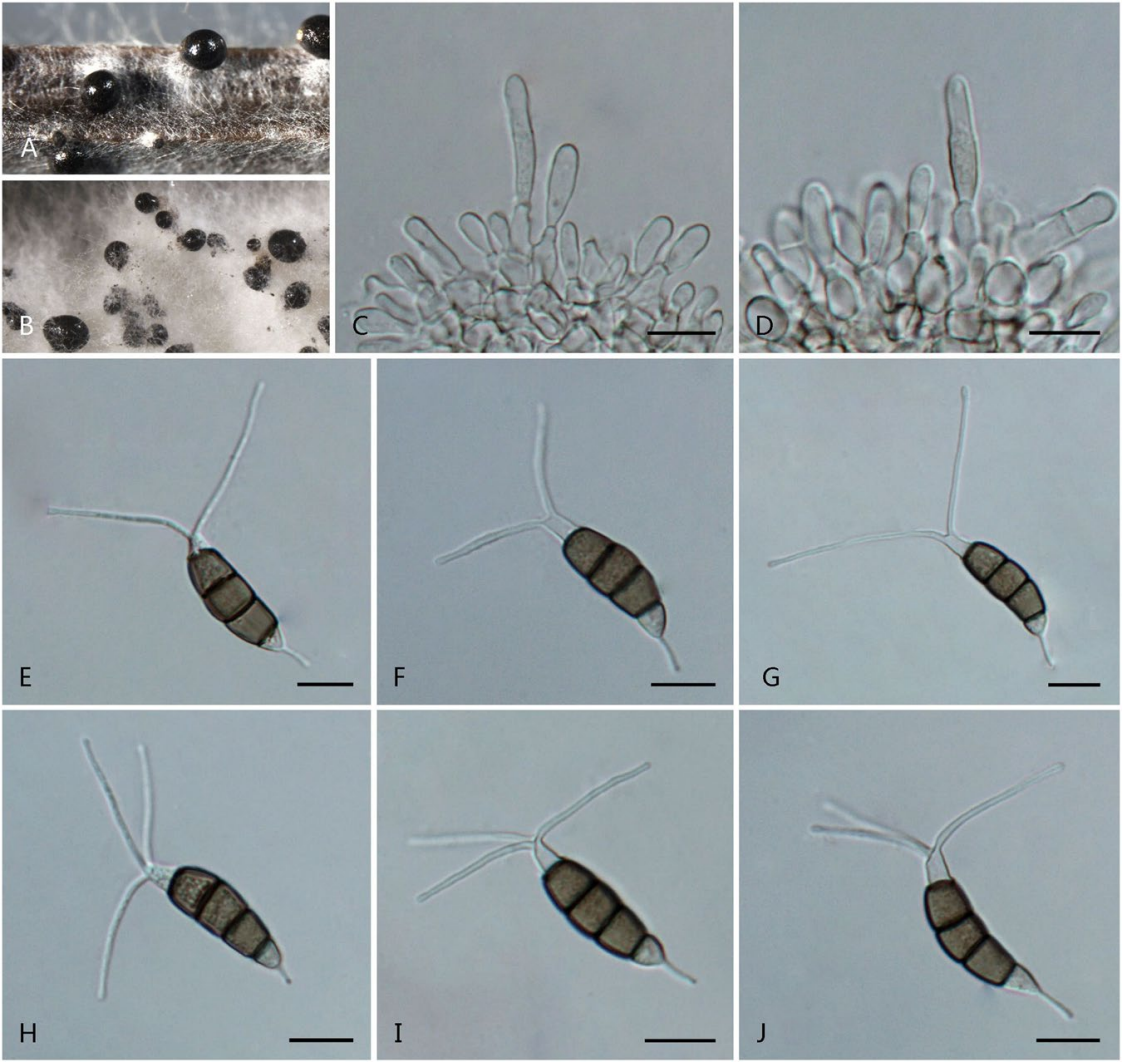
*Pseudopestalotiopsis ampullacea* (from ex-holotype culture CGMCC 3.18157 (= LC6618)). (**A**) Conidiomata sporulating on pine needles; (**B**) Conidiomata on PDA. (**C–D**) Conidiogenous cells and conidia. (**E–J**) Conidia. Scale bars = 10 μm.

***Pseudopestalotiopsis ampullacea*** F. Liu & L. Cai **sp. nov**. — MycoBank MB 818922 Fig. 14.

*Etymology*: referring to the ampulliform-shaped conidiogenous cells.

Conidiomata pycnidial in culture on PDA, globose or clavate, solitary or aggregated, semi-immersed to erumpent, dark brown to black, up to 500 μm diam.; exuding globose, dark brown to black conidial masses. Conidiophores reduced to conidiogenous cell. Conidiogenous cell discrete or integrated, ampulliform, hyaline, smooth or minutely verruculose, proliferating 1–3 time percurrently, 8.5–20 × 3.5–5 μm. Conidia fusoid, ellipsoid, straight to slightly curved, 4-septate, 21–31.5 × 6.5–9 μm (av. ± SD = 26 ± 2.8 × 7.7 ± 0.7 μm); basal cell conic, hemispherical or obconic with a truncate base, hyaline or pale brown, rugose and thin–walled, 2.5–5.5 μm long; three median cells doliiform, 13.5–19.5 μm (av. ± SD = 17.2 ± 1.4 μm) long, wall minutely verruculose, concoloured, septa darker than the rest of cell and conidium constructed at septum (second cell from base, 4.5–8.5 μm long; third cell 4.5–7 μm long; fourth cell 4–7 μm long); apical cell 3–5 μm long, hyaline, subcylindrical or obconic with a truncate base, thin-walled, slightly rugose; with 2–3 tubular apical appendages(mostly 3), arising from the apical crest, unbranched, filiform, 17–25 μm long; basal appendage single, tubular, centric, 3.5–7 μm long.

Culture characteristics: Colonies on PDA attaining 80 mm diam. after 7 d at 25 °C, with lobate edge, whitish, with moderate aerial mycelia on the surface with black, gregarious conidiomata; reverse whitish in colour.

Materials examined: China, Jiangxi Province, Yangling National Froest Park, on *Lauraceae*, 5 Sep. 2013, *Y.H. Gao*, living culture LC4479; Yunnan Province, Xishuangbanna, Jing Mai, on *Camellia sinensis*, 17 Apr. 2015, *F. Liu*, *HMAS 247056* (holotype), **ex-holotype** living culture CGMCC 3.18157 (= LC6618).

Notes: *Pseudopestalotiopsis ampullacea* is phylogenetically closely related to *Ps. indica* and *Ps. chinensis* (Fig. 5), but differs in producing shorter apical appendages (17–25 μm vs. 30–40 μm in *Ps. indica*, 24–41 μm in *Ps. chinensis*) and further from *Ps. indica* by its shorter conidia (21–31.5 μm vs. 31.5–37 μm).

**Figure 15:**
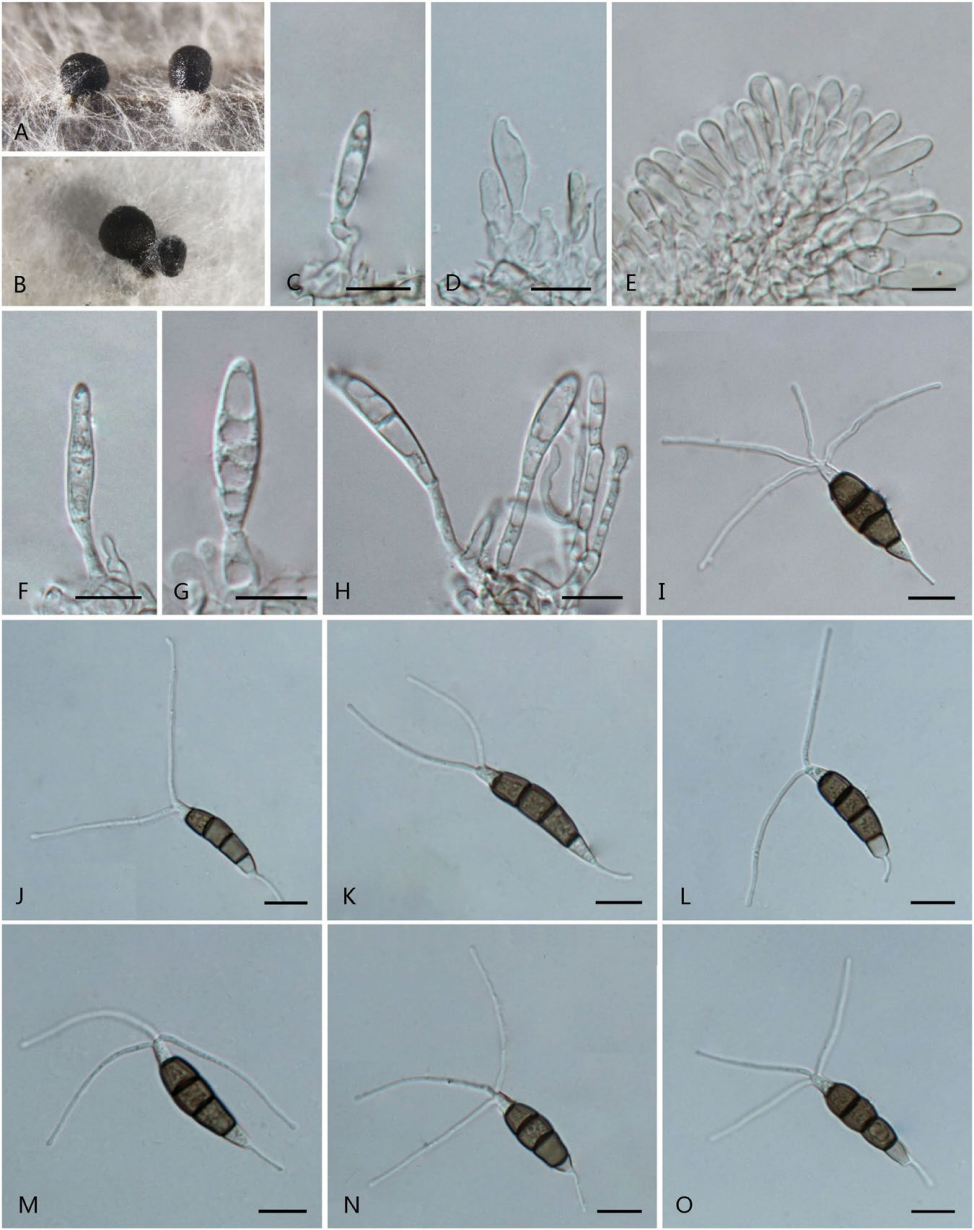
*Pseudopestalotiopsis camelliae-sinensis* (**A–E**, **J**–**O**) from ex-holotype culture CGMCC 3.18155 (= LC3490), (**F–I)** from LC6677. (**A**) Conidiomata sporulating on pine needle. (**B**) Conidiomata sporulating on PDA. (**C–H**) Conidiogenous cells and conidia. (**I–O**) Conidia. Scale bars = 10 μm.

***Pseudopestalotiopsis camelliae-sinensis*** F. Liu & L. Cai **sp. nov**. — MycoBank MB 818924 Fig. 15.

*Etymology*: named after the Latin name of the host, *Camellia sinensis*.

Conidiomata pycnidial in culture on PDA, globose or clavate, aggregated, semi-immersed to erumpent, dark brown to black, up to 700 μm diam; exuding globose, dark brown to black conidial masses. Conidiophores reduced to conidiogenous cells; when present, 1–2-septate, unbranched, or irregularly branched, hyaline, thin-walled. Conidiogenous cells discrete or integrated, ampulliform, subcylindrical or nearly globose, hyaline, smooth or minutely verruculose, 10–18 × 3–6 μm. Conidia fusoid, ellipsoid, straight to slightly curved, 4-septate, 23–34 × 5.5–9 μm (av. ± SD = 29.3 ± 2.5 × 7.6 ± 0.9 μm); basal cell conic or obconic with a truncate base, hyaline, rugose and thin-walled, 3.5–6.5 μm long; three median cells doliiform or subcylindrical, 16–21.5 μm (av. ± SD = 19.2 ± 1.3 μm) long, wall minutely verruculose, concoloured, septa darker than the rest of cell and conidium constructed at septum (second cell from base, 5–8.5 μm long; third cell 4–7 μm long; fourth cell 5–8 μm long); apical cell 3.5–6 μm long, hyaline, subcylindrical or obconic with a truncate base, thin-walled, slightly rugose; with 2–3 tubular apical appendages (mostly 3), rarely 4, arising from the apical crest, unbranched, filiform, 24–49.5 μm (av. ± SD = 36.8 ± 6.4 μm) long; basal appendage single, tubular, centric or uncentred, 5.5–9.5 μm long.

Culture characteristics: Colonies on PDA attaining 70–80 mm diam after 7 d at 25 °C, with lobate edge, whitish to pale honey-coloured, moderate aerial mycelia on the surface with black, gregarious conidiomata; reverse whitish to pale honey.

Materials examined: China, Fujian Province, Zhangzhou, on *Camellia sinensis*, Nov. 2012, *L. Cai*, LC3009; ibid. LC3021; ibid. LC3020; ibid. LC3022; ibid. LC3023; Guangxi Province, Guilin, on *C. sinensis*, Sep. 2013, *T.W. Hou*, *HMAS 247054* (holotype), **ex-holotype** living culture CGMCC 3.18155 (= LC3490); ibid. LC3487; Hangzhou, on *C. sinensis*, 4 Oct. 2013, *F. Liu*, LC3571; Yunnan Province, Xishuangbanna, Da Zhai, on *C. sinensis*, 19 Apr. 2015, *F. Liu*, LC6677.

Notes: This species was nomenclaturely proposed as *Pseudopestalotiopsis ignota* ^4^ (Page 3), but in other parts of the same paper, cited as *Ps. camelliae*. The provided IF registration number (IF 551719) also pointed to *Ps. ignota* (http://www.indexfungorum.org/names/NamesRecord.asp? RecordID = 551719). Index Fungorum provided a nomenclature comment as “*Typographical error in name where formally introduced (Pseudopestalotiopsis ignota Maharachch., L.D. Guo & K.D. Hyde, sp. nov.) but cited correctly (Pseudopestalotiopsis camelliae) elsewhere. Incorrect identifier cited, see Art. 42.1*”. Based on Art. 40.1 and 42.1 of International Code of Nomenclature for algae, fungi and plants, the name *Ps. camelliae* is invalid. We therefore propose a new species here.

**Figure 16:**
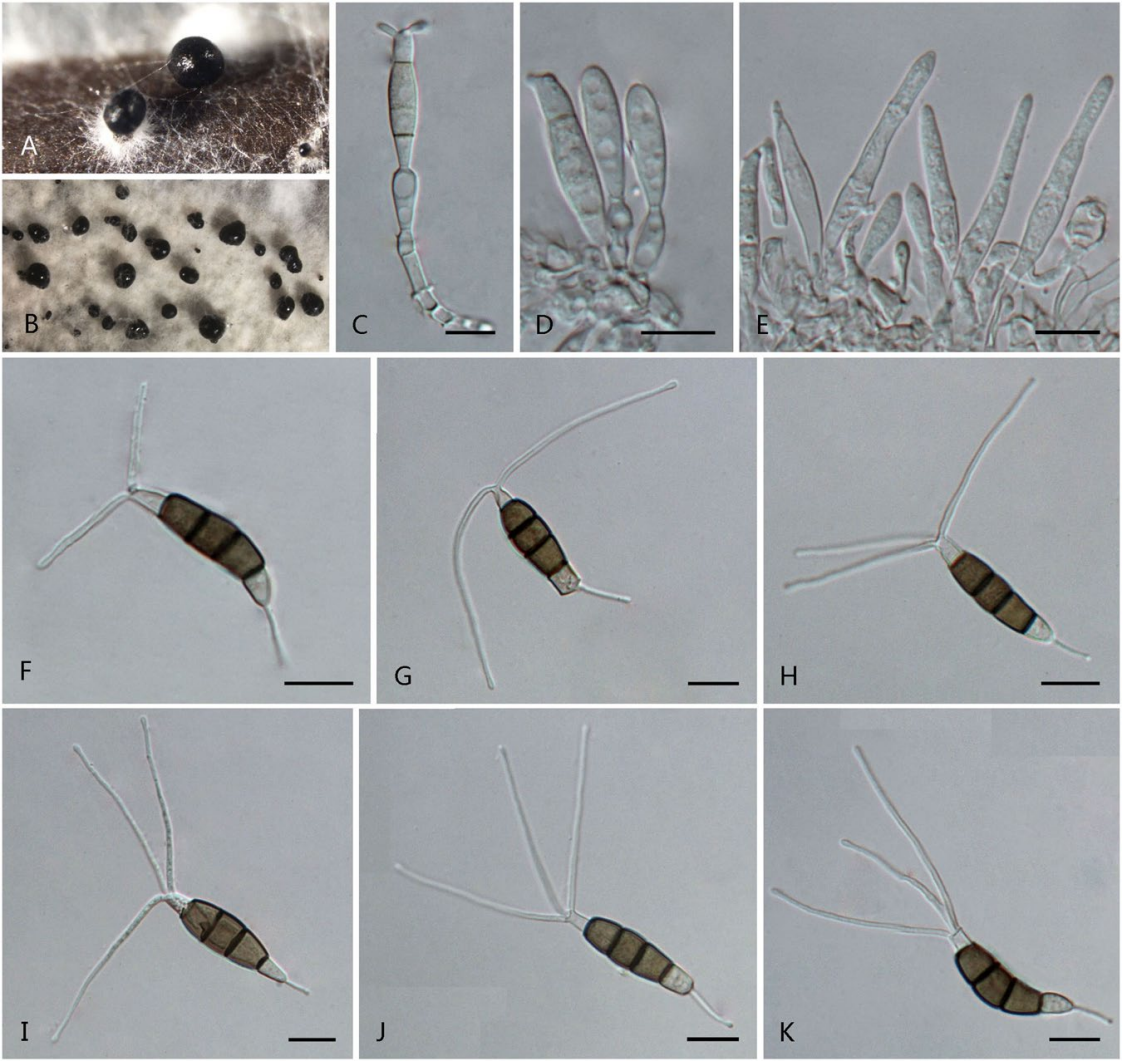
*Pseudopestalotiopsis chinensis* (from ex-holotype culture CGMCC 3.18152 (= LC3011)). (**A**) Conidiomata sporulating on pine needle. (**B**) Conidiomata sporulating on PDA. (**C–E**) Conidiogenous cells and conidia. (**F–K**) Conidia. Scale bars = 10 μm.

***Pseudopestalotiopsis chinensis*** F. Liu & L. Cai **sp. nov**. — MycoBank MB 818923 Fig. 16.

*Etymology*: named after the country where it was first collected, China.

Conidiomata pycnidial in culture on PDA, globose or clavate, aggregated or scattered, semi-immersed to erumpent, dark brown to black, up to 450 μm diam; exuding globose, dark brown to black conidial masses. Conidiophores indistinct, often reduced to conidiogenous cell. Conidiogenous cell discrete or integrated, clavate, cylindrical or subcylindrical, hyaline, smooth, thin-walled, 14–30 × 3–5.5 μm. Conidia fusoid, ellipsoid, straight to slightly curved, 4-septate, 25.5–35.5 × 6–9 μm (av. ± SD = 29.8 ± 2.6 × 7.4 ± 0.8 μm); basal cell conic, obconic or cylindrical with a truncate base, hyaline or slightly brown, versicoloured and thin-walled, 4.5–8 μm long; three median cells doliiform, 16.5–22.5 μm (av. ± SD = 19 ± 1.8 μm) long, wall minutely verruculose or rugose, concoloured, darker brown, septa darker than the rest of cell (second cell from base 5.5–8.5 μm long; third cell 4.5–7 μm long; fourth cell 5.5–8 μm long); apical cell 3.5–6 μm long, hyaline, conic or subcylindrical, thin–walled, slightly rugose; with 2–3 tubular apical appendages (mostly 3), arising from the apical crest, unbranched, filiform, 24–41 μm long; basal appendage single, tubular, centric, 5–12 μm long.

Culture characteristics: Colonies on PDA attaining 60 mm diam after 7 d at 25 °C, with entire edge, whitish, with moderate aerial mycelia on the surface with black, gregarious conidiomata; reverse whitish in colour.

Materials examined: China, Fujian Province, Zhangzhou, on *Camellia sinensis*, Nov. 2012, *L. Cai*, *HMAS 247051* (holotype), **ex-holotype** living culture CGMCC 3.18152 (= LC3011); ibid. LC3012; Yunnan Province, Xishuangbanna, Man Nai, on *C. sinensis*, 19 Apr. 2015, *F. Liu*, LC6629; Lao Man Sa, on *C. sinensis*, 19 Apr. 2015, *F. Liu*, LC6711; Tian Ba, on healthy leaves of *C. sinensis*, 21 Apr. 2015, *F. Liu*, LC6306; Gao Shan, on *Camellia* sp., 21 Apr. 2015, *F. Liu*, LC6695.

Notes: Although two supported subclades were recognised within the clade of *Ps. chinensis* (Fig. 5), we regarded them as geographic populations due to only two base pairs differences in sequenced loci and their indistinguishable morphological characters. *Pseudopestalotiopsis chinensis* is morphologically comparable to its closely related species *Ps. indica* on the shape and length of conidiogenous cells and conidia, but differs in the number of apical appendages (2–3 in *Ps. chinensis* vs. 3–4 in *Ps. indica*).

## Discussion

When segregating *Pestalotiopsis* into three genera, Maharachchikumbura *et al*.^18^ stated that, *Neopestalotiopsis* can be easily distinguished from *Pseudopestalotiopsis* and *Pestalotiopsis* by its versicolourous median cells and indistinct conidiophores, and *Pseudopestalotiopsis* differs from *Pestalotiopsis* by generally dark coloured concolourous median cells with indistinct conidiophores. Here, addition of more isolates into this group revealed several exceptions. For example, strain LC6285 nesting in *Neopestalotiopsis* produces concoloured median cells and distinct conidiophores (Supplementary Fig. S10), and a newly proposed species in *Pseudopestalotiopsis*, *Ps. camelliae-sinensis*, also produces distinct conidiophores (Fig. 15). Unfortunately our current sampling is insufficient to reasonably resolve the morphological circumscriptions of these genera.

Conidial appendages appear to be highly taxonomically informative at the species level^23, 24^ and have been widely used in *Pestalotiopsis* taxonomy^5, 18, 23, 25, 26^. The comparable characters of apical appendages include the length, number, shape, branch pattern and position^18^. We summarised the characters of *Pestalotiopsis* species, including apical and basal appendages, conidiogenous cells and conidia in Supplementary Table S1, and found that many species are distinct in the basal appendages, i.e. very short (normally shorter than 3 μm, e.g. *P. arenga*, *P. papuana*, *P. licualacola*), very long (normally longer than 10 μm, e.g. *P. brassicae*, *P. yunnanensis*), lack (i.e. *P. camelliae*, *P. furcate*, *P. yanglingensis*), branched (i.e. *P. brachiata*, *P. aggestorum*), more than 1 appendages (i.e. *P. biciliata*, *P. brachiata*, *P. dilucida*, *P. kenyana*). These morphological groupings showed a certain correspondence to the phylogeny, but clear-cut relationships could not be recognised.

Maharachchikumbura *et al*.^16^ selected ITS, TEF and TUB2 from 10 loci (ACT, CAL, GS, GAPDH, ITS, LSU, 18 S nrDNA, RPB1, TEF and TUB2) to resolve *Pestalotiopsis* through the comparison of morphological and sequence data. Phylogenetic analyses based on the combined ITS, TEF and TUB2 were then used to delimit species in *Neopestalotiopsis*, *Pestalotiopsis* and *Pseudopestalotiopsis*, and since then at least 92 new species/combinations have been introduced^e.g.^ refs 17, 18, 20, 21, 27–30. However, in the gene trees of *Neopestalotiopsis* in previous studies^18, 30^ and this study (Fig. 1), the overall branch-lengths were notably short and the support values were relatively low. Further studies of *Neopestalotiopsis* are therefore required to reveal whether the less informative loci lead to the poorly resolved phylogram, or if these poorly resolved branches are indicative of populations rather than species.

## Materials and Methods

### Sample collection and fungal isolation

Diseased and healthy tissues of tea plants (*Camellia sinensis*) and other *Camellia* spp. were collected from 26 locations in eight provinces in China (Fujian, Guangxi, Guizhou, Jiangxi, Sichuan, Tibet, Yunnan, Zhejiang) and one location in New Zealand (Mt. Albert). Pathogenic and endophytic fungi were isolated using single spore isolation and tissue isolation methods as described in Liu *et al*.^2^.

A total of 124 *Pestalotiopsis-*like strains were obtained, and all of them were deposited in the LC culture collection (personal culture collection of Lei Cai housed in the Institute of Microbiology, Chinese Academy of sciences) (Supplementary Table S2). Type specimens and ex-type living cultures of new species from this study were deposited in the Mycological Herbarium, Institute of Microbiology, Chinese Academy of Sciences, Beijing, China (HMAS), and China General Micriobiological Culture Collection centre (CGMCC), respectively.

### Morphology

Morphological descriptions were made for isolates cultivated on 2% potato dextrose agar (PDA; Difco). Conidiomatal development was observed on synthetic nutrient-poor agar (SNA) amended with double-autoclaved pine needles placed onto the agar surface. Cultures were incubated at room temperature (c. 25 °C) under near UV light with a 12 h photoperiod for 7 d. Microscopic preparations were made in distilled water, and at least 30 measurements per structure were noted and observed with a Nikon Eclipse 80i microscope using differential interference contrast (DIC) illumination. Taxonomic descriptions and nomenclature were deposited in MycoBank^31^.

### DNA extraction, PCR amplification and sequencing

Total genomic DNA was extracted from fungal mycelia using CTAB method. Four loci were amplified and sequenced for nucleotide sequence comparisons, including large subunit ribosomal DNA (LSU), the internal transcribed spacer regions and intervening 5.8 S nrRNA gene (ITS), the partial beta-tubulin (TUB2) and translation elongation factor 1-alpha (TEF). The primer pairs LR0R/LR5^32, 33^, ITS1/ITS4^34^, T1/Bt2b^35, 36^, and EF1-728F/EF2^37, 38^ were used for amplification and sequencing for these loci respectively. PCR amplification protocols were performed as described by Maharachchikumbura *et al*.^16^. Purification and sequencing of PCR amplifications were carried out by the Omegagenetics Company, Beijing, China. MEGA v.6.06 was used to obtain consensus sequences from DNA sequences generated from forward and reverse primers. All novel sequences obtained in this study were deposited in NCBI’s GenBank database (Supplementary Table S2).

### Species delimitation

LSU sequences of isolates from *Camellia sinensis* and other *Camellia* spp. generated in this study were supplemented with additional sequences obtained from ex-type cultures and other related strains of *Pestalotiopsis*-like species from GenBank (Supplementary Table S2). Sequences alignment was performed with MAFFT v.7, and was manually improved with MEGA v.6.06. After isolates were assigned to appropriate genera based on LSU sequence alignment using Maximum Likelihood (ML) method, multiple sequences analyses were then performed for each genus based on ITS, TUB2 and TEF. Bayesian inference (BI) and ML methods were implemented in this study. Bayesian analyses were performed using MrBayes v.3.2.2^39^ as outlined by Liu *et al*.^40^ and the best-fit evolutionary models for each locus were estimated in MrModeltest v.2.3 using the Akaike Information Criterion (AIC)^41^. ML analyses were performed using RAxML v.7.0.3^42^ with 1000 replicates under the GTR-GAMMA model.

## Electronic supplementary material

Supplementary info
