# Supplementary material for: Pestalotiopsis and allied genera from Camellia, with description of 11 new species from China
Source: Sci Rep. 2017 Apr 13;7:866. doi: 10.1038/s41598-017-00972-5 (PMC5429834; doi:10.1038/s41598-017-00972-5)
Supplement: Supplementary file 1 — Supplementary info [file 41598_2017_972_MOESM1_ESM.pdf]

Pestalotiopsis and allied genera from _Camellia_, with description of 11 new species from China

Fang Liu[1], Lingwei Hou[1,2], Mubashar Raza[1,2], Lei Cai[1,2*]

[1]Chinese Academy of Sciences, Institute of Microbiology, State Key Laboratory of Mycology, Beijing, 100101, China
[2]University of Chinese Academy of Sciences, Beijing 100049, China

*Corresponding author: cail@im.ac.cn

Supplementary Fig. S1 Phylogenetic tree of *Neopestalotiopsis* calculated with maximum likelihood analysis on the ITS dataset by running RAxML v.7.0.3. The RAxML bootstrap support values (> 50%) are displayed at the nodes.

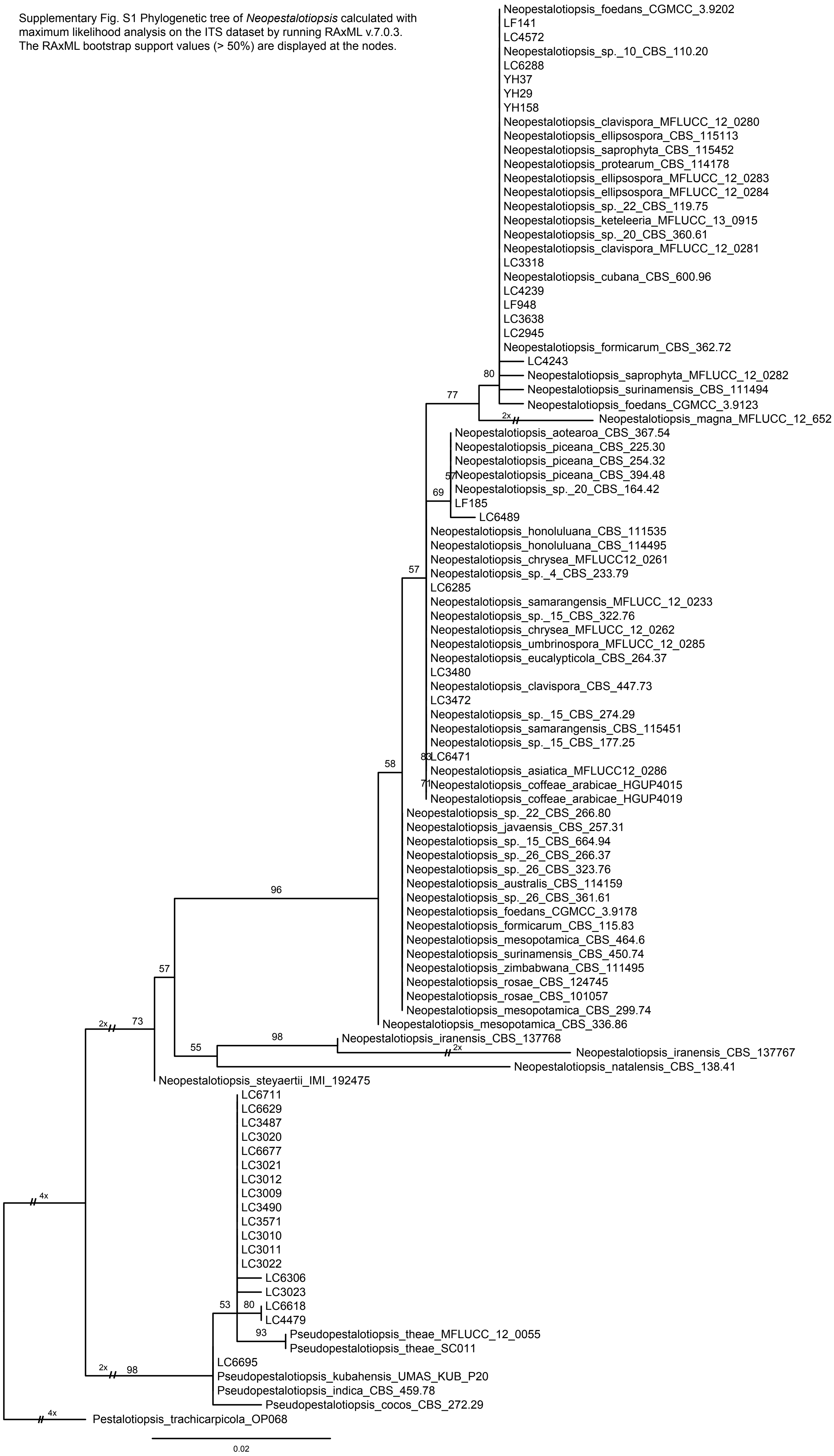

Supplementary Fig. S2 Phylogenetic tree of Neopestalotiopsis calculated with maximum likelihood analysis on the TEF dataset by running RAxML v.7.0.3. The RAxML bootstrap support values (> 50%) are displayed at the nodes.

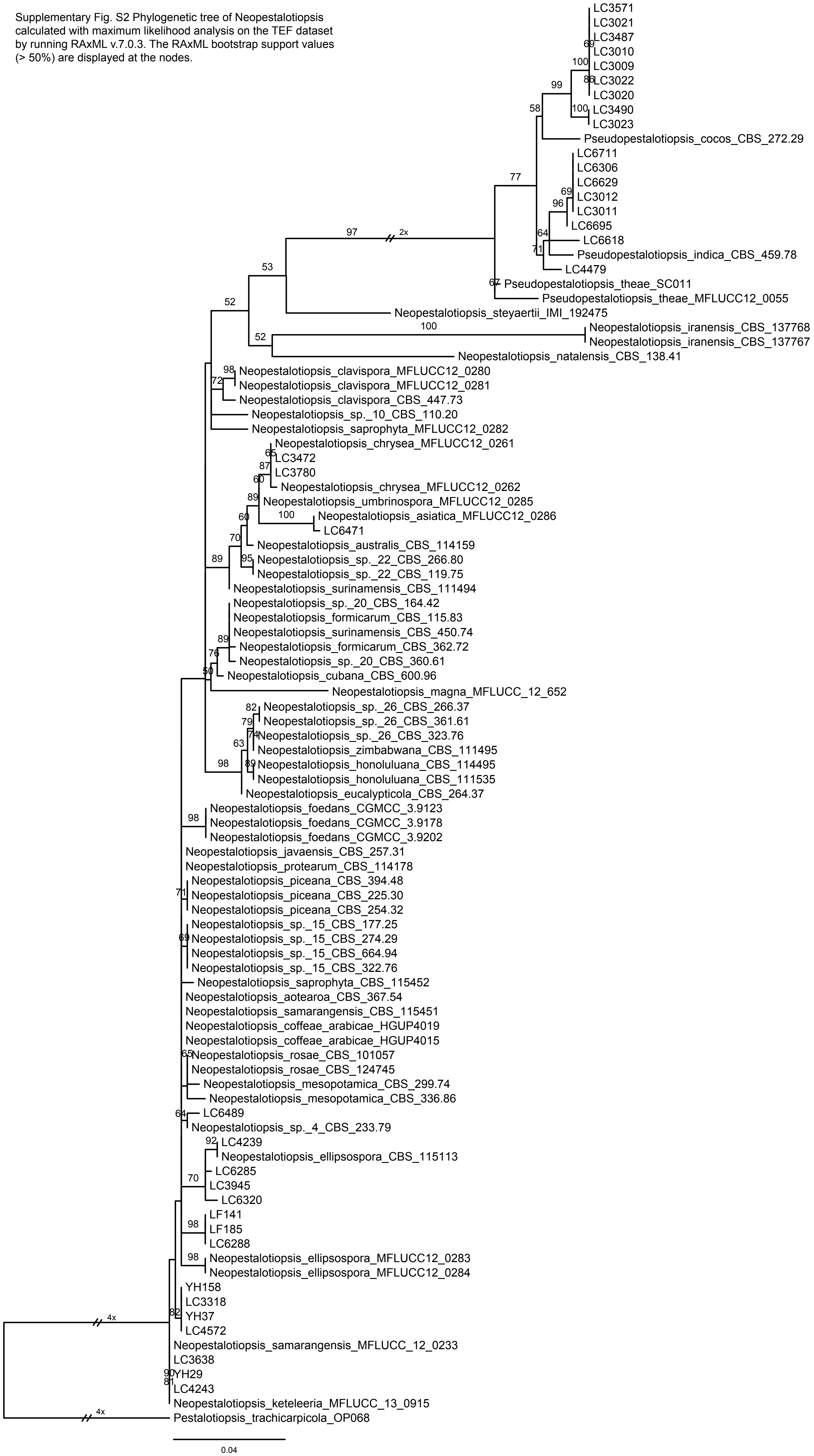

Supplementary Fig. S3 Phylogenetic tree of Neopestalotiopsis calculated with maximum likelihood analysis on the TUB2 dataset by running RAxML v.7.0.3. The RAxML bootstrap support values (> 50%) are displayed at the nodes.

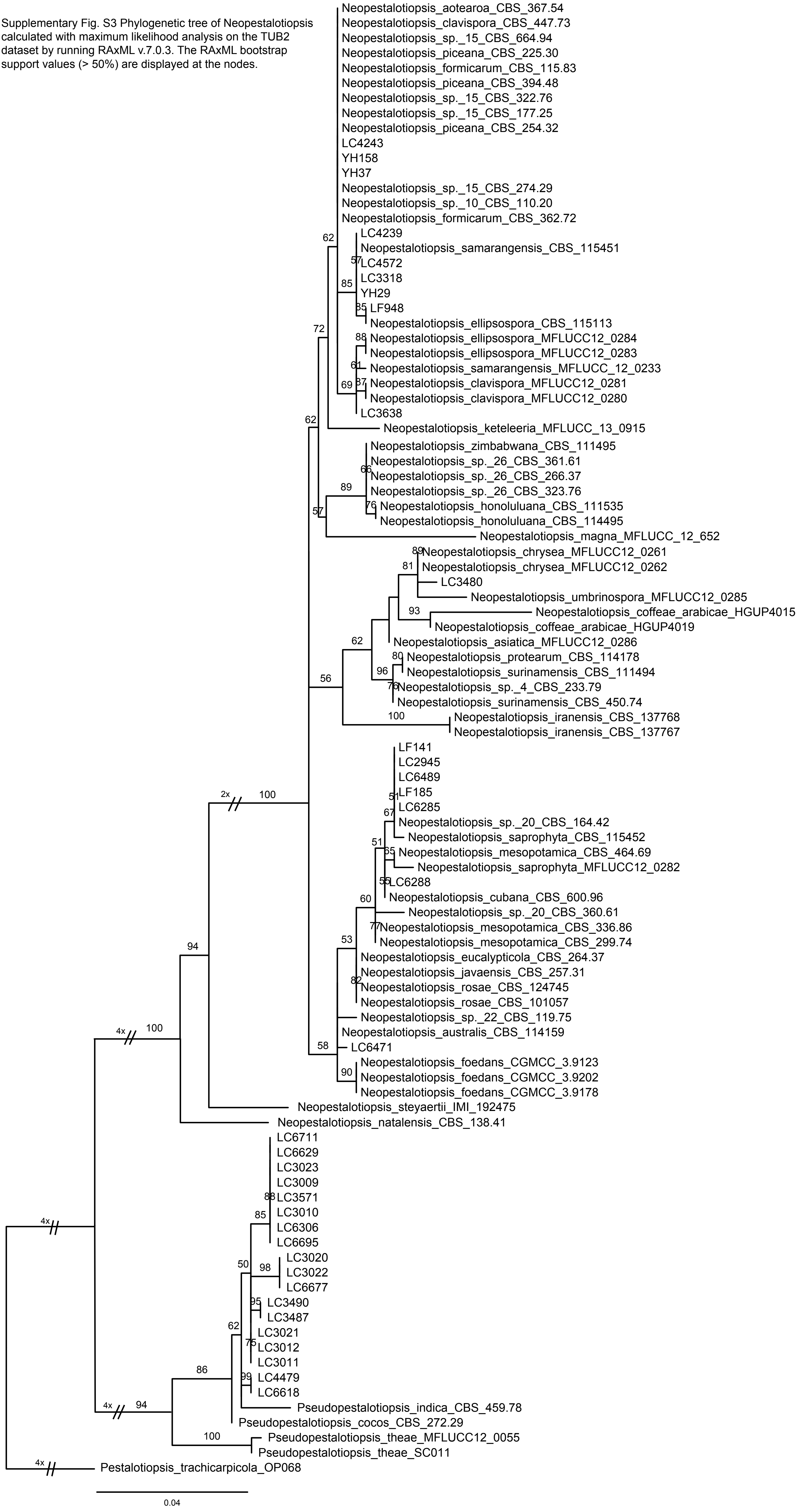

Supplementary Fig. S4 Phylogenetic tree of Pestalotiopsis calculated with maximum likelihood analysis on the ITS dataset by running RAxML v.7.0.3. The RAxML bootstrap support values (> 50%) are displayed at the nodes.

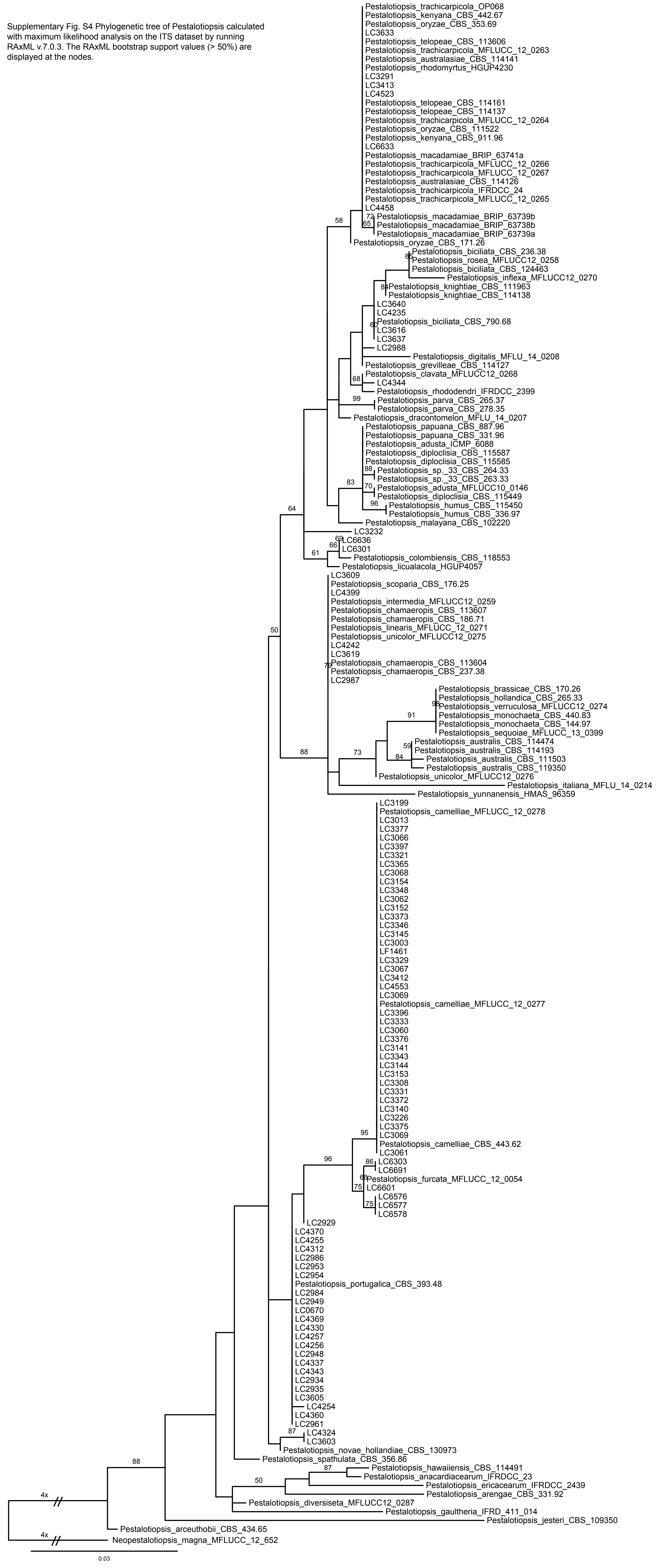

Supplementary Fig. S5 Phylogenetic tree of Pestalotiopsis calculated with maximum likelihood analysis on the TEF dataset by running RAxML v.7.0.3. The RAxML bootstrap support values (> 50%) are displayed at the nodes.

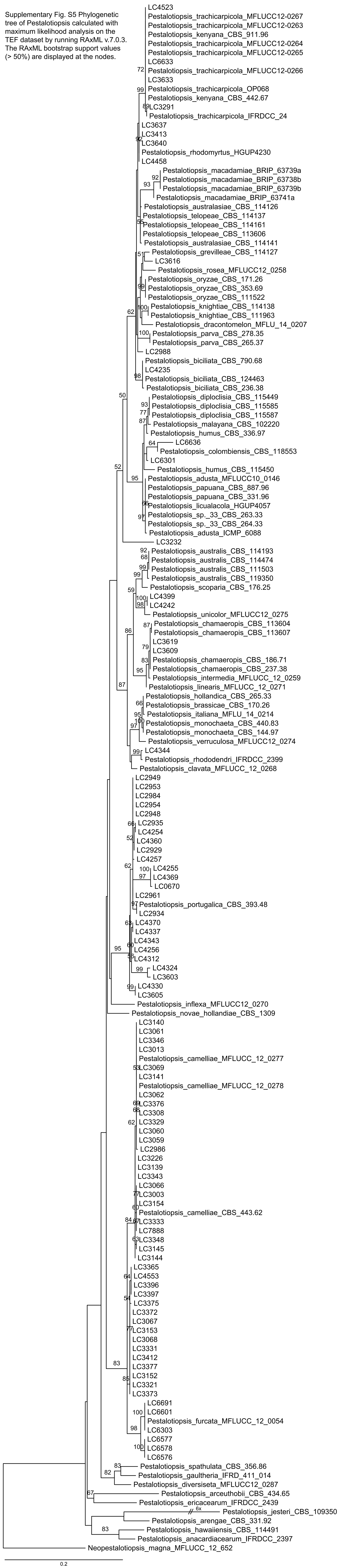

Supplementary Fig. S6 Phylogenetic tree of Pestalotiopsis calculated with maximum likelihood analysis on the TUB2 dataset by running RAxML v.7.0.3. The RAxML bootstrap support values (> 50%) are displayed at the nodes.

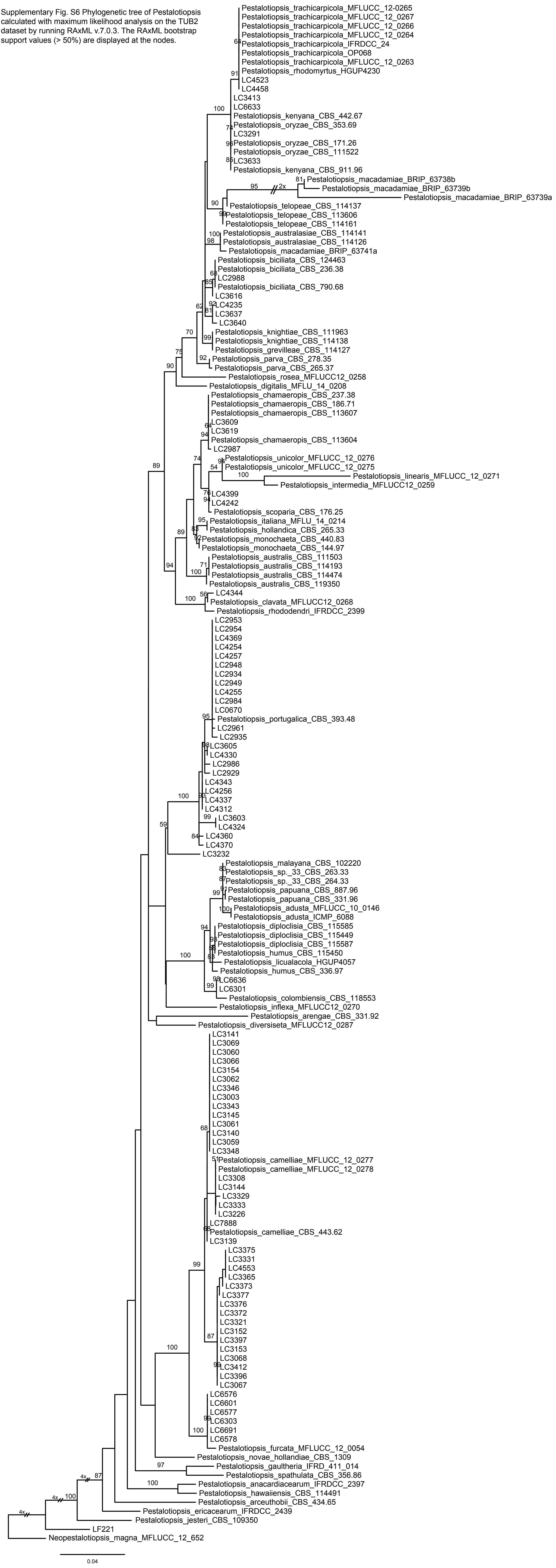

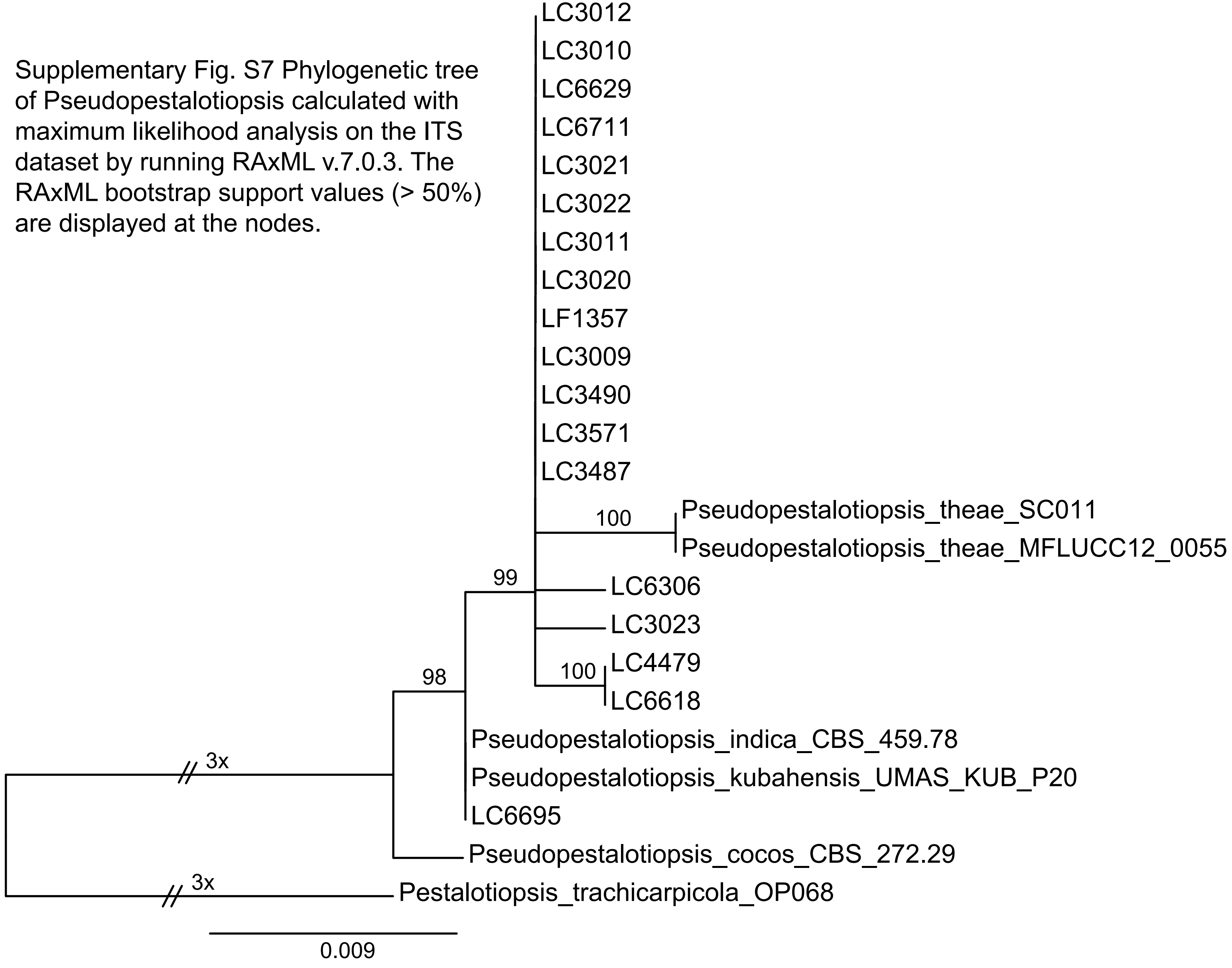

Supplementary Fig. S7 Phylogenetic tree of Pseudopestalotiopsis calculated with maximum likelihood analysis on the ITS dataset by running RAxML v.7.0.3. The RAxML bootstrap support values (> 50%) are displayed at the nodes.

Supplementary Fig. S8 Phylogenetic tree of N Pseudopestalotiopsis calculated with maximum likelihood analysis on the TEF dataset by running RAxML v.7.0.3. The RAxML bootstrap support values (> 50%) are displayed at the nodes.

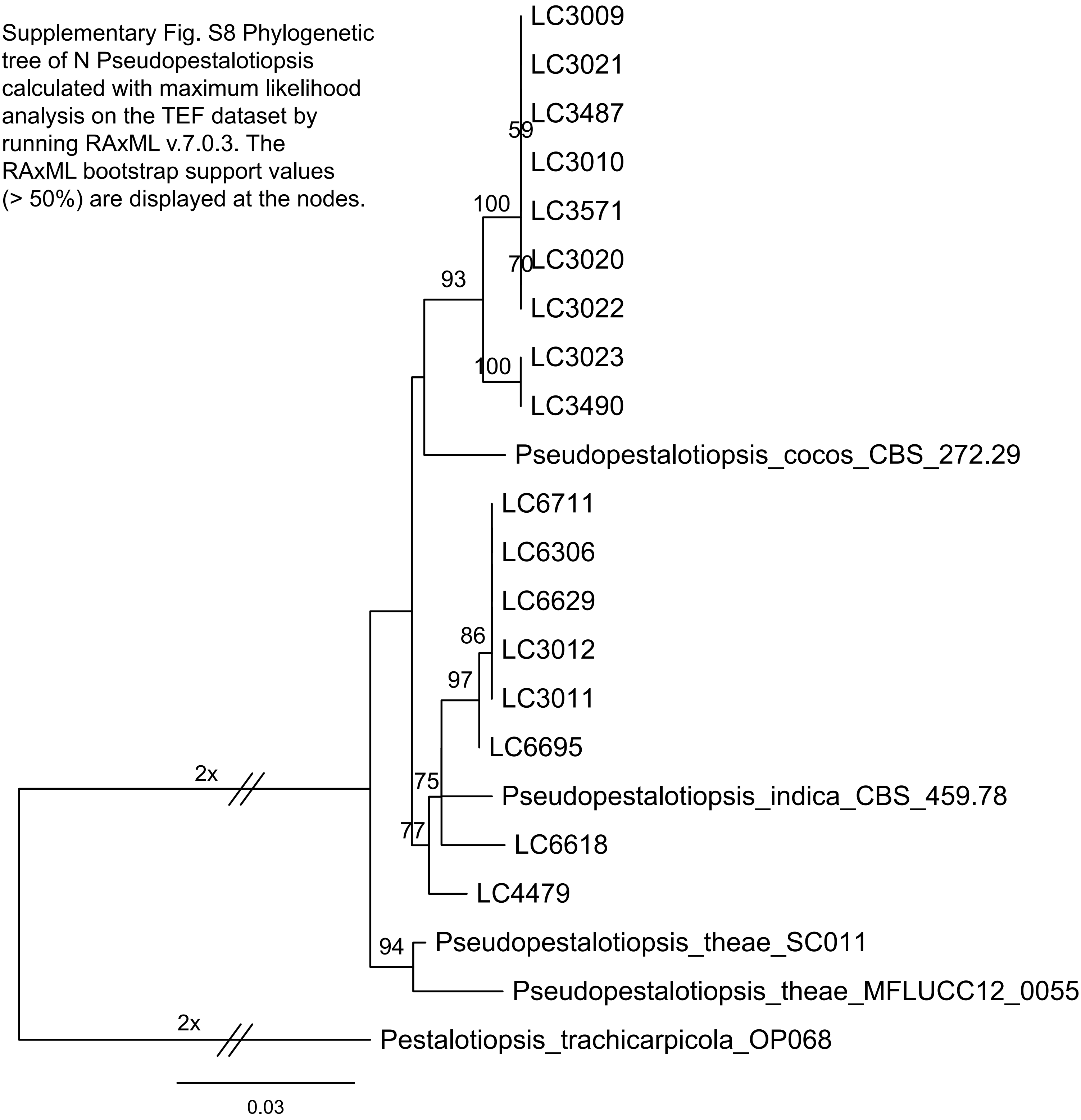

Supplementary Fig. S9 Phylogenetic tree of Pseudopestalotiopsis calculated with maximum likelihood analysis on the TUB2 dataset by running RAxML v.7.0.3. The RAxML bootstrap support values (> 50%) are displayed at the nodes.

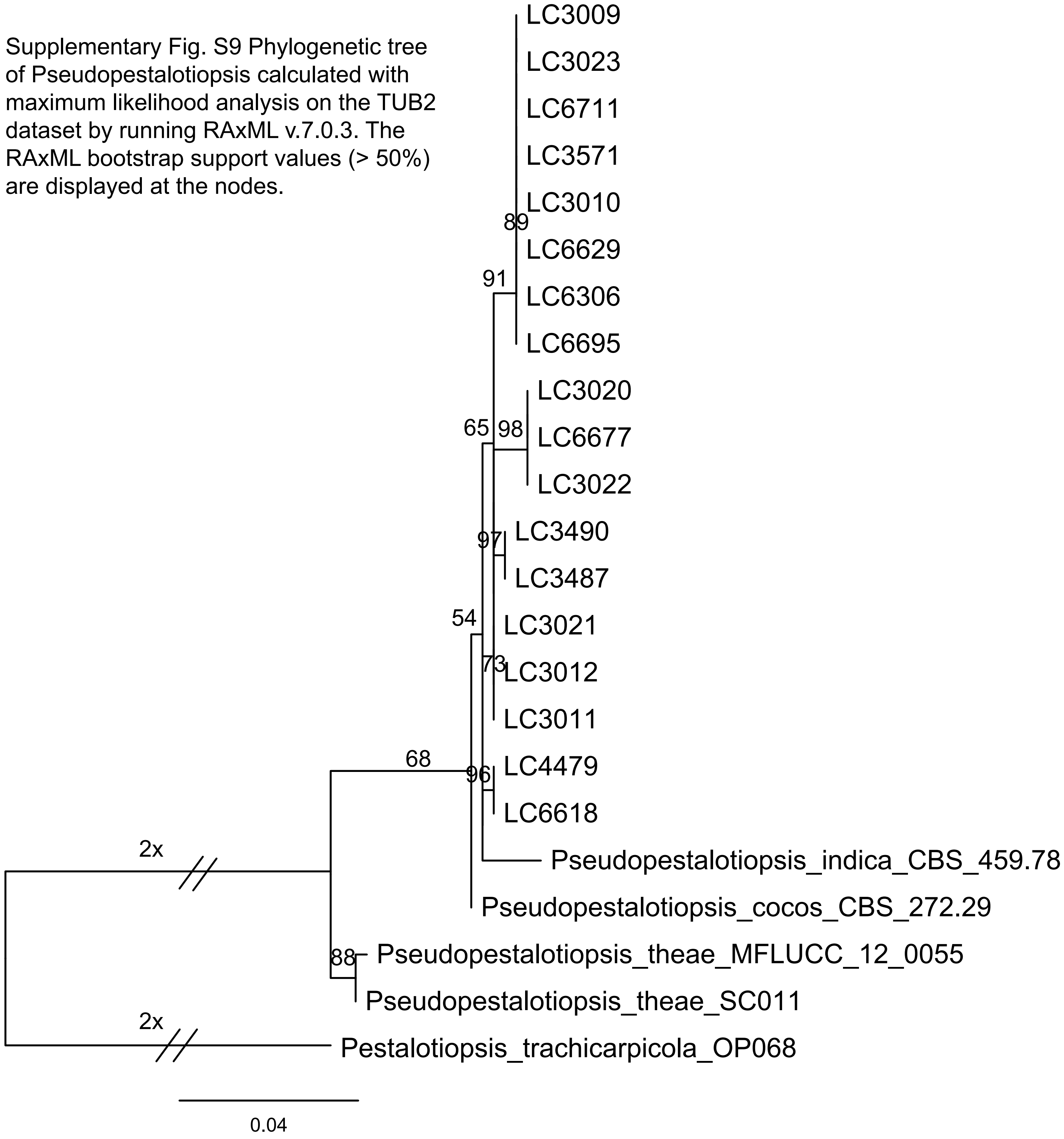

Supplementary Fig. S10 ***Neopestalotiopsis*** **sp.** (A–E, K–P from isolate LC6285; F–J, Q–S from isolate LC6471). A, F. Conidioma sporulating on pine needles; B, G. Conidioma on PDA. C–E, H–J. Conidiogenous cells and conidia. K–S. Conidia. Scale bars = 10 μm.

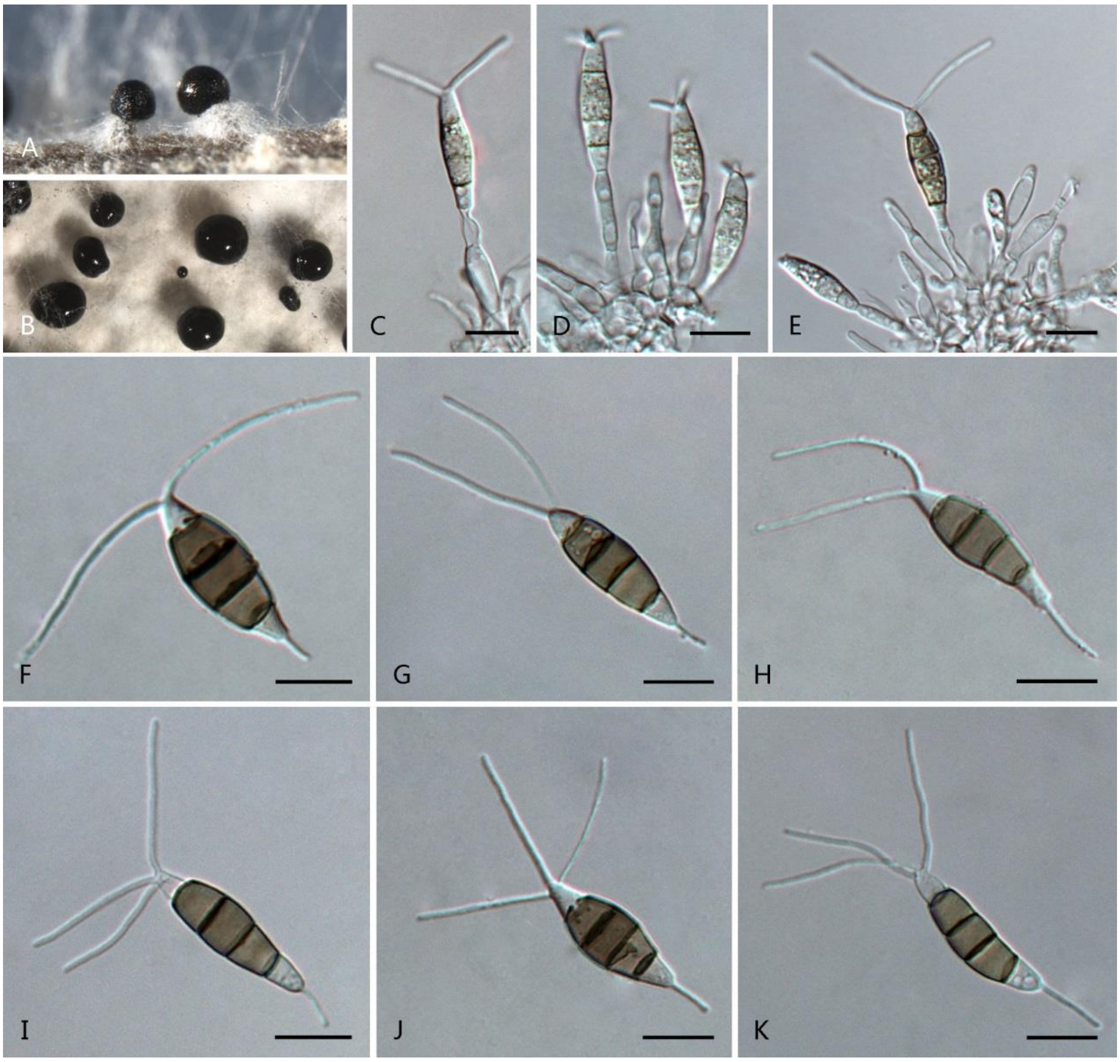

Supplementary Table S1 Summary of important morphological characters of species in *Pestalotiopsis*.

**Species**[a]	**Conidiogenous cells**	**Conidia size**	**No. of apical appendages**	**Branched or unbranched**	**Length of apical appendages**	**No. of basal appendages**	**Branched or unbranched**	**Length of basal appendages**
P. adusta[16]	filiform	17–20 × 5.2–6.6 µm (av. = 19 × 6 µm)	2–3	unbranched	6–14 µm (av. = 10 µm)	0–1	unbranched	
P. aggestorum[*]	ampulliform, clavate or subcylindrical, 6–14.7 × 2–5.5 µm	19–24.5 × 5–7 µm (av. ± SD = 21.5 ± 1.5 × 6.2 ± 0.6 µm)	2–3 (mostly 3)	rarely branched	18–28 µm	1–2	occasionally branched	5–14 µm
P. anacardiacearum[43]	branched or separate at the base	27–39 × 7–10 µm (av. = 32 × 9 µm)	2–3 (mostly 3)	unbranched	20–45 µm (av. = 33 µm)	1, rarely absent	unbranched	5–9 µm (av. = 7 µm)
P. arceuthobii[18]	subcylindrical, 3–12 × 1–3 µm; ampulliform to lageniform 3–10 × 2–6 µm	(21–)22–25.5(–26) × 6.5–8(–8.5) µm (av. ± SD = 24.4 ± 1.3 × 7.2 ± 0.5 µm)	2–3 (mostly 3)	unbranched	(10–)11–14.5(–16) µm (av. ± SD = 12.8 ± 1.0 µm)	1	unbranched	3–6 µm
P. arengae[18]	ampulliform to lageniform, 3–15 × 3–10 µm	(24–)25–32(–33) × 7–9.5(–10) µm (av. ± SD = 27.6 ± 2 × 8 ± 0.4 µm)	2–3 (mostly 3)	unbranched	(4–)4.5–11(–12) µm (av. ± SD = 7.3 ± 1.3 µm)	1	unbranched	1.5–3 µm
P. australasiae[18]	ampulliform or cylindrical, 15–50 × 3–9 µm	(23–)24.5–29(–31) × (6–)6.5–8(–8.5) µm (av. ± SD = 26 ± 1.4 × 7.5 ± 0.2 µm)	2–3	unbranched	(9–)10–15(–16) µm (av. ± SD = 12.6 ± 1.7 µm)	1	unbranched	2.5–4.5 µm
P. australis[18]	ampulliform or cylindrical, 20–60 × 2–6 µm	(26–)27–34(–36) × 7–8.5 µm (av. ± SD = 30.8 ± 2.1 × 7.7 ± 0.3 µm)	2–3 (mostly 3)	unbranched	(11–)12–20(–22) µm (av. ± SD = 15.5 ± 2.7 µm)	1	unbranched	3–7 µm
P. biciliata[18]	cylindrical to subcylindrical, 10–45 × 2–5 µm	(21–)22–28.5(–30) × (5.5–)6–7.5(–8) µm (av. ± SD = 25.3 ± 2 × 6.7 ± 0.3 µm)	2–3 (mostly 3)	unbranched	(6–)8–18(–20) µm (av. ± SD = 13.3 ± 3.2 µm)	2	unbranched	centric appendage 3–8 µm, excentric appendage 1–3 µm

P. brachiata[*]	ampulliform, 15–30 × 2–8 µm	23.5–25 × 6.5–8 µm (av. ± SD = 24.9 ± 1.4 × 7 ± 0.3 µm)	2–3 (mostly 2)	unbranched	16–28.5 µm (av. ± SD =22 ± 4.1 µm)	1–4	branched or unbranched	5.5–9.5 µm
P. brassicae[18]	cylindrical, 20–70 × 2–10 µm; ampulliform to lageniform, 4–10 × 3–8 µm	(29–)30–37(–40) × (8–)8.5–11(–11.5) µm (av. ± SD = 34 ± 2.1 × 9.7 ± 0.7 µm)	3–5 (mostly 4)	unbranched	(27–)28.5–48(–50) µm (av. ± SD = 37 ± 5 µm)	1	unbranched	10–25 µm
P. camelliae[5]	lageniform	27–33 × 7.5–9.3 µm (av. = 28.5 × 8.5 µm)	3–6	rarely branched	13.5–34 µm (av. = 23.8 µm)	0		
P. chamaeropis[18]	cylindrical, 20–50 × 2–5 µm	(21–)22.5–27(–28) × (6–)7–9(–9.5) µm (av. ± SD = 25.2 ± 1.3 × 8 ± 0.4 µm)	2–3 (mostly 3)	unbranched	(13–)14.5–23(–24) µm (av. ± SD = 18 ± 3.1 µm)	1	unbranched	4–8.5 µm
P. clavata[16]	ampulliform to lageniform	20–27 × 6.5–8 µm (av. = 22.6 × 7.3 µm)	2–3 (mostly 3)	unbranched	20–25 µm	1	unbranched	7–9 µm
P. colombiensis[18]	cylindrical, 10–50 × 2–8 µm	(19–)21–27(–28.5) × 5.5–7.5(–8) µm (av. ± SD = 24 ± 1.5 × 6.3 ± 0.5 µm)	2–3 (mostly 3)	unbranched	(11–)13–25(–28) µm (av. ± SD = 17.5 ± 3 µm)	1	unbranched	2–5 µm
P. digitalis[44]	ampulliform to lageniform	18–22 × 7–9 µm (av. = 20 × 8.2 µm	1–3 (mostly 2)	unbranched	8–17 µm (av. = 13 µm)	1	unbranched	4–7 µm
P. diploclisiae[18]	cylindrical to subcylindrical, 6–20 × 2–5 µm	(20–)22–26.5(–28) × 5–6.5(–7) µm (av. ± SD = 24 ± 1.3 × 5.7 ± 0.4 µm)	2–4 (mostly 3)	unbranched	(10–)13–19(–22) µm (av. ± SD = 16.6 ± 2.1 µm)	1	unbranched	3–8 µm
P. dilucida[*]	ampulliform, 7.5–20 × 2–4 µm	24.5–32 × 5.5–8.5 µm (av. ± SD = 27.3 ± 2.27 × 6.76 ± 0.91 µm)	2–5 (mostly 3)	unbranched or branched	(10–)16.5–39.5 µm (av. ± SD = 23.1 ± 5.6 µm)	1–2	unbranched	5.5–20(–28.5) µm
P. diversiseta[16]		27–34 × 5.5–8 µm (av. = 29.7 × 6.3 µm)	3–5 (rarely 2)	rarely branched	22–30 µm	1	unbranched	5–9 µm
P. dracontomelon[44]	cylindrical	18–23 × 5.5–7.5 µm (av. = 20 × 6.5 µm)	2–3	unbranched	11–20 µm (av. = 16 µm)	1	unbranched	2–7 µm

P. ericacearum[45]	cylindrical, lageniform or claviform	(15)16–20(21) × 5–9 µm (av. = 18 × 6.5 µm)	3–4 (mostly 3), unequal in length	unbranched	(19)20–43(45) µm (av. = 32 µm)	1	unbranched	2–9 µm (av. = 4 µm)
P. furcata[6]	lageniform	29–39 × 8.5–10.5 µm (av. = 35.5 × 9.7 µm)	5–9, unequal in length	branched	20–35 µm (av. = 27.7 µm)	0		
P. gaultheriae[45]	branched or unbranched at the base or above	(20)23–31(33) × 7–9.5 µm (av. = 26.4 × 8.6 µm)	3	unbranched	(13–)15–50(–54) µm (av. = 35 µm)	1, rarely absent	unbranched	2.5–4 µm
P. grevilleae[18]	cylindrical to subcylindrical, 5–25 × 2–8 µm	(21–)22.5–28(–29) × (7–)7.5–9(–9.5) µm (av. ± SD = 25.2 ± 1.2 × 8.2 ± 0.5 µm)	2–3 (mostly 3)	unbranched	(12–)14–26.5(–29) µm (av. ± SD = 19 ± 3 µm)	1	unbranched	3–8 µm
P. hawaiiensis[18]	cylindrical, 20–50 × 3–6 µm	(26–)27–34.5(–37) × (7–)7.5–10(–10.5) µm (av. ± SD = 31.6 ± 2 × 8.7 ± 0.6 µm)	2–3 (mostly 3)	unbranched	(14–)19–33(–36) µm (av. ± SD = 25.3 ± 4.1 µm)	1	unbranched	5–11 µm
P. hollandica[18]	cylindrical, tapering to a long, thin neck	(25–)25.5–33(–34) × 8.5–10(–10.5) µm (av. ± SD = 28 ± 2 × 9.4 ± 0.3 µm)	1–4	branched	20–40 µm (av. ± SD = 27 ± 1.5 µm)	1	unbranched	3–9 µm
P. humus[18]	cylindrical, 8–28 × 2–5 µm	(17–)18.5–22(–23) × 5–7(–7.5) µm (av. ± SD = 20 ± 1.4 × 6 ± 0.4 µm)	2–3	unbranched	(6–)6.5–12(–13) µm (av. ± SD = 9.0 ± 1.5 µm)	1	unbranched	2–5 µm
P. inflexa[16]	ampulliform to lageniform	24–31 × 6–9 µm (av. = 27 × 7.6 µm)	2–5 (mostly 3–4)	rarely branched	20–30 µm (av. = 24 µm)	1	unbranched	9–15 µm
P. intermedia[16]	filiform	24–28 × 5.5–6.5 µm (av. = 25.7 µm)	2–3 (rarely 4)	rarely branched	10–28 µm (av. = 18.5 µm), unequal	1	unbranched	6–10 µm
P. italiana[44]	subcylindrical or ampulliform to lageniform	26–35 × 8–11 µm (av. = 30 × 9.6 µm)	2–5 (mostly 3–4)	unbranched	20–40 µm (av. = 32 µm)	1	unbranched	6–10 µm (av. = 7 µm).

P. jesteri[18]	lageniform to subcylindrical, 5–20 × 3–7 µm	(21–)22.5–31(–34.5) × 7–9 µm (av. ± SD = 26.8 ± 3 × 8.2 ± 0.2 µm)	apical appendage single; lateral appendages 2–4, arising just above the septum separating the apical cell and upper median cell	unbranched	apical appendage 14–25 µm; lateral appendages 14–25 µm	1	unbranched	4–14 µm
P. jiangxiensis[*]	ampulliform, clavate, obclavate or cylindrical, 11.5–29.5 × 2–5.5 µm	22–29 × 6–9 µm (av. ± SD = 25.7 ± 1.7 × 7.3 ± 0.7 µm)	2–4 (mostly 3), inserted at different loci in the upper half of the apical cell	rarely branched	16.5–32 µm (av. ± SD = 22.4 ± 3.8 µm)	1	unbranched	6.5–19.5 µm
P. jinchanghensis[*]	spheriform, cylindrical, or clavate, hyaline, 5–12 × 2–7 µm	22–32 × 5.5–8.5 µm, (av. ± SD = 26.5 ± 2.5 × 6.5 ± 0.65 µm)	1–3 (mostly 2)	unbranched	15–33.5 µm long, (av. ± SD = 21.5 ± 4.2 µm)	1–2	unbranched	5.5–15.5 µm
P. kenyana[18]	lageniform to subcylindrical, 10–25 × 2–5 µm	(22–)23–28(–29) × 7–9 µm (av. ± SD = 25.5 ± 1.2 × 8 ± 0.4 µm)	2–3 (mostly 3)	unbranched	(8–)9–18(–20) µm (av. ± SD = 14 ± 3 µm)	2	unbranched	centric appendage 3–20 µm, eccentric appendage 1–4 µm
P. keteleeria[20]	ampulliform, 2.5-6 × 2-5 µm	18.5-24 × 7-9.5 µm (av. = 22 × 8.5 µm)	1-3 (mostly 3)	unbranched	7.5-21 µm (av. = 14 µm)	0, rarely 1	unbranched	1-3.5 µm (av. = 2 µm)

P. knightia[18]	ampulliform or lageniform	10–30 × 2–10 µm	2–4 (mostly 3)	unbranched	(8–)12–20(–23) µm (av. ± SD = 15 ± 3.9 µm)	1	unbranched	2.5–7.5 µm
P. licualicola[46]	filiform, 4–10 µm	16–20 × 3–5 µm (av. = 17.4 × 3.9 µm)	1–3 (mostly 1)	unbranched	4–9.5 µm (av. = 6.6 µm)	0–1	unbranched	very short
P. linearis[16]	ampulliform to lageniform	24–33 × 4.7–6 µm (av. = 29 × 5.5 µm)	2–3 (rarely 1)	unbranched	10–20 µm (av. = 15 µm), unequal in length	1 (rarely 2)	unbranched	4–7 µm
P. longiappendiculata[*]	ampulliform or cylindrical, 3.5–5.5 µm	28–34 × 7–10 µm (av. ± SD = 31.8 ± 2.0 × 8.3 ± 0.8 µm)	2–3	branched or unbranched	29.5–47.5 µm (av. ± SD = 37.5 ± 4.6 µm)	1	unbranched	3.5–9 µm
P. lushanensis[*]	ampulliform, clavate or subcylindrical, 8–40 × 4–6 µm	20–27 × 7.5–10 µm, (av. ± SD = 22.3 ± 1.9 × 8.6 ± 0.6 µm)	2–3 (mostly 3)	unbranched	17–26 µm (av. ± SD = 20.3 ± 2.9 µm)	1	unbranched	4–6.5 µm
P. malayana[18]	subcylindrical to ampulliform, 6–18 × 2–4 µm	(20.5–)22–29.5(–31) × 5–7.5 µm (av. ± SD = 25.6 ± 2 × 6.3 ± 0.4 µm)	1–3 (mostly 2)	unbranched	(11–)11.5–18.5(–19) µm (av. ± SD = 15.1 ± 1.4 µm)	1	unbranched	2–5 µm
P. monochaeta[18]	ampulliform to lageniform, 4–12 × 2–4 µm; cylindrical, 10–60 × 2–8 µm	(25–)27–40(–42) × 7–11(–11.5) µm (av. ± SD = 32.8 ± 3.5 × 9.6 ± 0.6 µm)	1	unbranched	(40–)43–67(–75) µm (av. ± SD = 51 ± 6 µm)	1	unbranched	6–14 µm
P. novae-hollandiae[18]	lageniform, 5–20 × 5–10 µm	(24–)25–31(–32) × (7.5–)8–10(–10.5) µm (av. ± SD = 28.1 ± 1.6 × 9 ± 0.7 µm)	3–9, arising at a different locus in the upper half of the cell	branched	(20–)22–44(–50) µm (av. ± SD = 31 ± 9 µm)	1	unbranched	2–5 µm
P. oryza[18]	ampulliform to lageniform, 10–25 × 3–7 µm	(23–)24.5–29(–30) × 6–8 µm (av. ± SD = 26.9 ± 1.4 × 7 ± 0.2 µm)	2–3 (mostly 3)	unbranched	(9–)18–27(–17) µm (av. ± SD = 12.9 ± 1.7 µm)	1	unbranched	3–6 µm
P. papuana[18]	lageniform to subcylindrical, 4–20 × 2–4 µm	(17–)18–22(–24) × 6–7.5 µm (av. ± SD	1–2	unbranched	1.5–7 µm (av. ± SD = 4.1 ± 1 µm)	1	unbranched	0.5–2 µm

		= 20.5 ± 1.5 × 6.7 ± 0.3 µm)						
P. parva[18]	cylindrical to subcylindrical, 5–18 × 2–4 µm	(16–)16.5–20(–21) × 5–7(–7.5) µm (av. ± SD = 18.3 ± 1.2 × 6.2 ± 0.5 µm)	2–3 (mostly 3)	unbranched	(6–)6.5–12(–13) µm (av. ± SD = 9.0 ± 1.9 µm)	1	unbranched	2–4 µm
P. portugalica[18]	cylindrical, 10–60 × 4–12 µm	(14.5–)15.5–20(–21.5) × 5–7 µm (av. ± SD = 17.9 ± 1.6 × 6.0 ± 0.5 µm)	1–3	unbranched or branched	(8–)9–18(–20) µm (av. ± SD = 14 ± 3 µm)	0 or 1	unbranched	1–4 µm
P. rhododendri[45]	filiform	(16–)18–27(–29) × 5–8 µm (av. = 21 × 7 µm)	3	unbranched	(5–)7–15(–18) µm (av. = 14 µm)	1	unbranched	2–6 µm (av. = 4 µm)
P. rhodomyrtus[28]	filiform	19.7–26.3 × 4.9–6.7 µm (av. = 23 × 5.76 µm	2–3, unequal in length	unbranched	7.5–14.9 µm (av. = 10.54 µm)	1	unbranched	2.8–4.9 µm (av. = 3.65 µm)
P. rosea[16]	ampulliform to lageniform	17.5–21.8 × 5.7–7 µm (av. = 19.2 × 6.2 µm)	1–3	branched	14–22 µm	1 (rarely absent)	unbranched	2–5.7 µm
P. scoparia[18]	cylindrical to subcylindrical, 10–30 × 2–4 µm	(22–)23.5–29(–31) × 6–8.5 µm (av. ± SD = 26.3 ± 2 × 7.4 ± 0.3 µm)	3–5	unbranched	(20–)23–35(–42) µm long (av. ± SD = 29.6 ± 4 µm)	1	unbranched	9–25 µm
P. spathulata[18]	ampulliform to lageniform or cylindrical, 5–40 × 2–8 µm	(24–)25–31(–32) × 7.5–9.5 µm (av. ± SD = 27.7 ± 2 × 8.6 ± 0.3 µm)	2–5, each inserted at a different locus in the upper half of the cell	branched	(17–)18–24(–25) µm (av. ± SD = 21.1 ± 1.7 µm)	1	unbranched	4–7 µm
P. telopeae[18]	ampulliform or lageniform, 5–15 × 2–9 µm	(24–)24.5–31(–32) × 6–8 µm, av. ± SD = 27 ± 1.5 × 7 ± 0.3 µm	2–4 (mostly 3)	unbranched	(7–)8–15(–16) µm (av. ± SD = 12.6 ± 1.7 µm)	1	unbranched	3.5–7 µm
P. trachicarpicola[16]	filiform	20–25 × 5.5–7.2 µm (av. = 23.5 × 6.5 µm)	2–3	unbranched	9–18 µm (av. = 13.6 µm)	1	unbranched	4–8 µm (av. = 6.3 µm)

P. unicolor[16]	ampulliform to lageniform	20–24.5 × 4–6 µm (av. = 22 × 5 µm)	2–3	unbranched	11–20 µm (av. = 17.5 µm), in unequal length	1 (rarely 2)	unbranched	4–10 µm
P. verruculosa[16]		28–35 × 9–11 µm (av. = 30.6 × 10.3 µm)	2–6 (mostly 3–4)	unbranched	25–40 µm (av. = 34 µm)		unbranched	8–12 µm
P. yanglingensis[*]	obpyriform, ampulliform, cylindrical or subcylindrical, 11.5–36 × 3–8 µm	25–36 × 7–11 µm (av. ± SD = 29.4 ± 1.8 × 8.9 ± 0.78 µm)	2–5 (mostly 3)	unbranched or branched	16–30 µm (av. ± SD = 25.3 ± 2.92 µm)	0		
P. yunnanensis[47]	lageniform to ampulliform or subcylindrical, 8–17.3 × 2.5–4.9 µm (av. = 12.8 × 3.3 µm).	26–33.8 × 6.5–8.2 µm (av. = 28.8 × 7.2 µm)	3–7 (mostly 4–5)	unbranched	19.5–40.3 µm (av. = 28.9 µm)	1	unbranched	8.7–22.9 µm (av. = 16.4 µm)

[a] The numbers after species names indicate the cited references where their morphological characters come from; [*] indicates the newly proposed species in this study.

Supplementary Table S2 Details of isolates included in the phylogenetic analyses.

Species	Culture	Substrate	Family	Location	LSU	ITS	TUB	TEF
Neopestalotiopsis aotearoa	CBS 367.54*	canvas	-	New Zealand	KM116247	KM199369	KM199454	KM199526
N. asiatica	MFLUCC 12-0286*	unidentified tree	-	China	-	JX398983	JX399018	JX399049
N. australis	CBS 114159*	*Telopea* sp.	Proteaceae	New South Wales	KM116252	KM199348	KM199432	KM199537
N. chrysea	MFLUCC 12-0261*	dead leaves	-	China	-	JX398985	JX399020	JX399051
	MFLUCC 12-0262	dead plant	-	China	-	JX398986	JX399021	JX399052
N. clavispora	CBS 447.73	decaying wood	-	Sri Lanka	KM116275	KM199374	KM199443	KM199539
	MFLUCC 12-0280	*Magnolia* sp.	Magnoliaceae	China	-	JX398978	JX399013	JX399044
	MFLUCC 12-0281*	*Magnolia* sp.	Magnoliaceae	China	-	JX398979	JX399014	JX399045
N. coffeae-arabicae	HGUP4015	*Coffea arabica*	Rubiaceae	China	-	KF412647	KF412641	KF412644
	HGUP4019*	*Coffea arabica*	Rubiaceae	China	-	KF412649	KF412643	KF412646
N. cubana	CBS 600.96*	leaf litter	-	Cuba	KM116253	KM199347	KM199438	KM199521
N. ellipsospora	CBS 115113	*Ardisia crenata*	Myrsinaceae	Hong Kong	KM116269	KM199343	KM199450	KM199544
	MFLUCC 12-0283*	plant litter	-	China	-	JX398980	JX399016	JX399047
	MFLUCC 12-0284	plant litter	-	Thailand	-	JX398981	JX399015	JX399046
N. eucalypticola	CBS 264.37*	*Eucalyptus globulus*	Myrtaceae	-	KM116256	KM199376	KM199431	KM199551
N. foedans	CGMCC 3.9178	*Neodypsis decaryi*	Arecaceae	China	-	JX398989	JX399024	JX399055
	CGMCC 3.9123*	Mangrove plant	-	China	-	JX398987	JX399022	JX399053
	CGMCC 3.9202	*Calliandra haematocephala*	Fabaceae	China	-	JX398988	JX399023	JX399054
N. formicarum	CBS 115.83	plant debris	-	Cuba	KM116255	KM199344	KM199444	KM199519
	CBS 362.72*	dead *Formicidae* (Ant)	-	Ghana	KM116248	KM199358	KM199455	KM199517
N. honoluluana	CBS 111535	*Telopea* sp.	Proteaceae	Hawaii	KM116263	KM199363	KM199461	KM199546
	CBS 114495*	*Telopea* sp.	Proteaceae	Hawaii	-	KM199364	KM199457	KM199548
N. iranensis	CBS 137767	*Fragaria ananassa*	Rosaceae	Iran	-	KM074045	KM074056	KM074053
	CBS 137768*	*Fragaria ananassa*	Rosaceae	Iran	-	KM074048	KM074057	KM074051

N. javaensis	CBS 257.31*	*Cocos nucifera*	Arecaceae	Java	-	KM199357	KM199437	KM199543
N. magna	MFLUCC 12-652*	*Pteridium* sp.	Dennstaedtiaceae	France	-	KF582795	KF582793	KF582791
N. mesopotamicum	CBS 299.74	*Eucalyptus* sp.	Myrtaceae	Turkey	KM116257	KM199361	KM199435	KM199541
	CBS 336.86*	*Pinus brutia*	Pinaceae	Iraq	KM116271	KM199362	KM199441	KM199555
	CBS 464.69	*Achras sapota*	Sapotaceae	India	KM116244	KM199353	KM199436	-
N. natalensis	CBS 138.41*	*Acacia mollissima*	Fabaceae	South Africa	KM116279	KM199377	KM199466	KM199552
N. piceana	CBS 225.30	*Mangifera indica*	Anacardiaceae	-	KM116270	KM199371	KM199451	KM199535
	CBS 254.32	*Cocos nucifera*	Arecaceae	Sulawesi	KM116267	KM199372	KM199452	KM199529
	CBS 394.48*	*Picea* sp.	Pinaceae	UK	KM116266	KM199368	KM199453	KM199527
N. protearum	CBS 114178*	*Leucospermum cuneiforme* cv. 'Sunbird'	Proteaceae	Zimbabwe	JN712564	JN712498	KM199463	KM199542
N. rosa	CBS 101057*	*Rosa* sp.	Rosaceae	New Zealand	KM116245	KM199359	KM199429	KM199523
	CBS 124745	*Paeonia suffruticosa*	Paeoniaceae	USA	KM116272	KM199360	KM199430	KM199524
N. samarangensis	CBS 115451	unidentified tree	-	Hong Kong	-	KM199365	KM199447	KM199556
	MFLUCC 12-0233*	*Syzygium samarangense*	Myrtaceae	Thailand	-	JQ968609	JQ968610	JQ968611
N. saprophyta	CBS 115452	*Litsea rotundifolia*	Lauraceae	Hong Kong	KM116251	KM199345	KM199433	KM199538
	MFLUCC 12-0282*	*Magnolia* sp.	Magnoliaceae	China	-	JX398982	JX399017	JX399048
Neopestalotiopsis sp.	**LC2945 = LF153**	*Camellia* sp., pathogen	Theaceae	China, Sichuan, Chengdu	KX895047	KX894924	KX895256	KX895142
Neopestalotiopsis sp.	**LC3318 = LF540**	*C. sinensis*, pathogen	Theaceae	China, Jiangxi, Yangling	KX895057	KX894964	KX895296	KX895181
Neopestalotiopsis sp.	**LC3472 = LF697**	*C. sinensis*, pathogen	Theaceae	China, Jiangxi, Ganzhou	KX895059	KX894982	-	KX895199
Neopestalotiopsis sp.	**LC3480 = LF707**	*C. sinensis*, pathogen	Theaceae	China, Jiangxi, Ganzhou	KX895060	KX894983	KX895314	KX895200
Neopestalotiopsis sp.	**LC3638 = LF867**	*Camellia* sp., pathogen	Theaceae	China, Jiangxi, Ganzhou	KX895064	KX894994	KX895325	KX895211
Neopestalotiopsis sp.	**LC6285 = LF1289**	*C. sinensis*, pathogen	Theaceae	China, Yunnan, Xishuangbanna, Jing Mai	KX895041	KX895013	KX895346	KX895232
Neopestalotiopsis sp.	**LC6288 = LF1293**	*C. sinensis*, pathogen	Theaceae	China, Yunnan, Xishuangbanna, Jin Chang He	KX895042	KX895014	KX895347	KX895233
Neopestalotiopsis sp.	**LC6320 = LF948**	*C. sinensis*, pathogen	Theaceae	China, Guizhou	KX895065	KX895018	KX895351	KX895237

Neopestalotiopsis sp.	**LC6471 = LF1103**	*C. sinensis*, endophyte	Theaceae	China, Yunnan, Xishuangbanna, Tian Ba	KX895036	KX895019	KX895352	KX895238
Neopestalotiopsis sp.	**LC6489 = LF1122**	*C. sinensis*, endophyte	Theaceae	China, Yunnan, Xishuangbanna, Da Qi Shu	KX895037	KX895020	KX895353	KX895239
Neopestalotiopsis sp. Clade IV	CBS 233.79	*Crotalaria juncea*	Fabaceae	India	KM116249	KM199373	KM199464	KM199528
Neopestalotiopsis sp. Clade X	CBS 110.20	-	-	-	KM116250	KM199342	KM199442	KM199540
Neopestalotiopsis sp. Clade XV	CBS 177.25	*Dalbergia* sp.	Fabaceae	-	KM116246	KM199370	KM199445	KM199533
	CBS 274.29	*Cocos nucifera*	Arecaceae	Java	KM116261	KM199375	KM199448	KM199534
	CBS 322.76	*Camellia* sp.	Theaceae	France	KM116259	KM199366	KM199446	KM199536
	CBS 664.94	*Cocos nucifera*	Arecaceae	Netherlands	KM116254	KM199354	KM199449	KM199525
Neopestalotiopsis sp. Clade XX	CBS 164.42	dune sand	-	France	KM116268	KM199367	KM199434	KM199520
	CBS 360.61	*Cinchona* sp.	Rubiaceae	Guinea	KM116260	KM199346	KM199440	KM199522
Neopestalotiopsis sp. Clade XXII	CBS 119.75	*Achras sapota*	Sapotaceae	India	KM116265	KM199356	KM199439	KM199531
	CBS 266.80	*Vitis vinifera*	Vitaceae	India	KM116264	KM199352	-	KM199532
Neopestalotiopsis sp. Clade XXVI	CBS 266.37	*Erica* sp.	Ericaceae	Germany	KM116273	KM199349	KM199459	KM199547
Neopestalotiopsis sp. Clade XXVI	CBS 323.76	*Erica gracilis*	Ericaceae	France	KM116262	KM199350	KM199458	KM199550
	CBS 361.61	*Cissus* sp.	Vitaceae	Netherlands	KM116274	KM199355	KM199460	KM199549
N. steyaertii	IMI 192475*	*Eucalyptus viminalis*	Myrtaceae	Australia	KM116285	KF582796	KF582794	KF582792
N. surinamensis	CBS 111494	*Protea eximia*	Proteaceae	Zimbabwe	JX556250	JX556232	KM199462	KM199530
	CBS 450.74*	*Elaeis guineensis*	Arecaceae	Suriname	KM116258	KM199351	KM199465	KM199518
N. umbrinospora	MFLUCC 12-0285*	unidentified tree	-	China	-	JX398984	JX399019	JX399050
N. zimbabwana	CBS 111495*	*Leucospermum cunciforme* cv. 'Sunbird'	Proteaceae	Zimbabwe	JX556249	JX556231	KM199456	KM199545
Pestalotiopsis adusta	ICMP 6088*	on refrigerator door PVC gasket	-	Fiji	-	JX399006	JX399037	JX399070
P. adusta	MFLUCC 10-146	*Syzygium* sp.	Myrtaceae	Thailand	-	JX399007	JX399038	JX399071

P. aggestorum	**LC6301 = LF1308***	*C. sinensis*, pathogen	Theaceae	China, Yunnan, Xishuangbanna, Jing Mai	KX895129	KX895015	KX895348	KX895234
	LC8186 = LF2076	*C. sinensis*, pathogen	Theaceae	China, Yunnan, Xishuangbanna, Jing Mai	-	KY464140	KY464160	KY464150
	LC8187 = LF2077	*C. sinensis*, pathogen	Theaceae	China, Yunnan, Xishuangbanna, Jing Mai	-	KY464141	KY464161	KY464151
P. anacardiacearum	IFRDCC 2397*	*Mangifera indica*	Anacardiaceae	China	-	KC247154	KC247155	KC247156
P. arceuthobii	CBS 434.65*	*Arceuthobium campylopodum*	Santalaceae	USA	KM116243	KM199341	KM199427	KM199516
P. arenga	CBS 331.92*	*Arenga undulatifolia*	Arecaceae	Singapore	KM116207	KM199340	KM199426	KM199515
P. australasia	CBS 114126*	*Knightia* sp.	Proteaceae	New Zealand	KM116218	KM199297	KM199409	KM199499
	CBS 114141	*Protea* sp.	Proteaceae	New South Wales	KM116203	KM199298	KM199410	KM199501
P. australis	CBS 111503	*Protea neriifolia* x *susannae* cv. *"Pink Ice"*	Proteaceae	South Africa	KM116200	KM199331	KM199382	KM199557
	CBS 114193*	*Grevillea* sp.	Proteaceae	New South Wales	KM116197	KM199332	KM199383	KM199475
	CBS 114474	*Protea neriifolia* x *susannae* cv. *"Pink Ice"*	Proteaceae	South Africa	KM116220	KM199334	KM199385	KM199477
	CBS 119350	*Brabejum stellatifolium*	Proteaceae	South Africa	KM116209	KM199333	KM199384	KM199476
P. biciliata	CBS 124463*	*Platanus x hispanica*	Platanaceae	Slovakia	KM116224	KM199308	KM199399	KM199505
	CBS 236.38	*Paeonia* sp.	Proteaceae	Italy	KM116214	KM199309	KM199401	KM199506
	CBS 790.68	*Taxus baccata*	Taxaceae	Netherlands	KM116235	KM199305	KM199400	KM199507
P. brachiata	**LC2988 = LF196***	*Camellia* sp., endophyte	Theaceae	China, Sichuan, Chengdu	-	KX894933	KX895265	KX895150
	LC8188 = LF2078	*Camellia* sp., endophyte	Theaceae	China, Sichuan, Chengdu	-	KY464142	KY464162	KY464152
	LC8189 = LF2079	*Camellia* sp., endophyte	Theaceae	China, Sichuan, Chengdu	-	KY464143	KY464163	KY464153
P. brassicae	CBS 170.26*	*Brassica napus*	Brassicaceae	New Zealand	-	KM199379	-	KM199558
P. camelliae	CBS 443.62	*Camellia sinensis*	Theaceae	Turkey	KM116225	KM199336	KM199424	KM199512
	**LC3003 = LF211**	*C. sinensis*, pathogen	Theaceae	China, Fujian, Zhangzhou	KX895074	KX894934	KX895266	KX895151
	**LC3059 = LF267**	*C. sinensis*, pathogen	Theaceae	China, Jiangxi, Yangling	KX895075	KX894944	KX895276	KX895161
	**LC3060 = LF268**	*C. sinensis*, pathogen	Theaceae	China, Jiangxi, Yangling	KX895076	KX894945	KX895277	KX895162
	**LC3061 = LF269**	*C. sinensis*, pathogen	Theaceae	China, Jiangxi, Yangling	KX895077	KX894946	KX895278	KX895163

	**LC3062 = LF270**	*C. sinensis*, pathogen	Theaceae	China, Jiangxi, Yangling	KX895078	KX894947	KX895279	KX895164
	**LC3066 = LF274**	*C. sinensis*, pathogen	Theaceae	China, Jiangxi, Yangling	KX895079	KX894948	KX895280	KX895165
	**LC3069 = LF277**	*C. sinensis*, pathogen	Theaceae	China, Jiangxi, Yangling	KX895082	KX894951	KX895283	KX895168
	**LC3139 = LF347**	*C. sinensis*, pathogen	Theaceae	China, Jiangxi, Yangling	KX895083	KX894952	KX895284	KX895169
	**LC3140 = LF348**	*C. sinensis*, pathogen	Theaceae	China, Jiangxi, Yangling	KX895084	KX894953	KX895285	KX895170
	**LC3141 = LF349**	*C. sinensis*, pathogen	Theaceae	China, Jiangxi, Yangling	KX895085	KX894954	KX895286	KX895171
	**LC3144 = LF352**	*C. sinensis*, pathogen	Theaceae	China, Jiangxi, Yangling	KX895086	KX894955	KX895287	KX895172
	**LC3145 = LF353**	*C. sinensis*, pathogen	Theaceae	China, Jiangxi, Yangling	KX895087	KX894956	KX895288	KX895173
	**LC3154 = LF363**	*C. sinensis*, pathogen	Theaceae	China, Jiangxi, Yangling	KX895090	KX894959	KX895291	KX895176
	**LC3226 = LF438**	*C. sinensis*, pathogen	Theaceae	China, Jiangxi, Yangling	KX895091	KX894960	KX895292	KX895177
	**LC3308 = LF530**	*C. sinensis*, pathogen	Theaceae	China, Jiangxi, Yangling	KX895094	KX894963	KX895295	KX895180
	**LC3329 = LF551**	*C. sinensis*, pathogen	Theaceae	China, Jiangxi, Yangling	KX895096	KX894966	KX895298	KX895183
	**LC3333 = LF555**	*C. sinensis*, pathogen	Theaceae	China, Jiangxi, Yangling	KX895098	KX894968	KX895300	KX895185
	**LC3343 = LF565**	*C. sinensis*, pathogen	Theaceae	China, Jiangxi, Yangling	KX895099	KX894969	KX895301	KX895186
	**LC3346 = LF568**	*C. sinensis*, pathogen	Theaceae	China, Jiangxi, Yangling	KX895100	KX894970	KX895302	KX895187
	**LC3348 = LF570**	*C. sinensis*, pathogen	Theaceae	China, Jiangxi, Yangling	KX895101	KX894971	KX895303	KX895188
	**LC3376 = LF598**	*C. sinensis*, pathogen	Theaceae	China, Jiangxi, Yangling	KX895105	KX894976	KX895308	KX895193
	**LC7888 = LF1461**	*C. sinensis*, pathogen	Theaceae	China, Tibet, Lulang	KX895137	KX895033	KX895366	KX895251
	MFLUCC 12-0278	*Camellia japonica*	Theaceae	China	KM116284	JX399011	JX399042	JX399075
	MFLUCC 12-0277*	*Camellia japonica*	Theaceae	China	-	JX399010	JX399041	JX399074
P. chamaeropis	CBS 113604	-	-	-	KM116201	KM199323	KM199389	KM199471
	CBS 113607	-	-	-	KM116211	KM199325	KM199390	KM199472
	**LC2987 = LF195**	*Camellia* sp., endophyte	Theaceae	China, Sichuan, Chengdu	-	KX894932	KX895264	-
	**LC3609 = LF838**	*Camellia* sp., pathogen	Theaceae	China, Jiangxi, Lushan Botanical Garden	KX895112	KX894989	KX895320	KX895206
	**LC3619 = LF848**	*Camellia* sp., pathogen	Theaceae	China, Jiangxi, Ganzhou	KX895114	KX894991	KX895322	KX895208

	CBS 186.71*	*Chamaerops humilis*	Arecaceae	Italy	KM116210	KM199326	KM199391	KM199473
	CBS 237.38	-	-	Italy	KM116217	KM199324	KM199392	KM199474
P. clavata	MFLUCC 12-0268*	*Buxus* sp.	Buxaceae	China	-	JX398990	JX399025	JX399056
P. colombiensis	CBS 118553*	*Eucalyptus eurograndis*	Myrtaceae	Colombia	KM116222	KM199307	KM199421	KM199488
P. digitalis	MFLU 14–0208*	*Digitalis purpurea*	Plantaginaceae	New Zealand	-	KP781879	KP781883	-
P. diploclisia	CBS 115449	*Psychotria tutcheri*	Rubiaceae	Hong Kong	KM116215	KM199314	KM199416	KM199485
	CBS 115585	*Diploclisia glaucescens*	Menispermaceae	Hong Kong	KM116213	KM199315	KM199417	KM199483
	CBS 115587*	*Diploclisia glaucescens*	Menispermaceae	Hong Kong	KM116242	KM199320	KM199419	KM199486
P. dilucida	**LC3232 = LF444***	*C. sinensis*, pathogen	Theaceae	China, Jiangxi, Yangling	KX895092	KX894961	KX895293	KX895178
	LC8184 = LF2074	*C. sinensis*, pathogen	Theaceae	China, Jiangxi, Yangling	-	KY464138	KY464158	KY464148
	LC8185 = LF2075	*C. sinensis*, pathogen	Theaceae	China, Jiangxi, Yangling	-	KY464139	KY464159	KY464149
P. diversiseta	MFLUCC 12-0287*	*Rhododendron* sp.	Ericaceae	China	-	JX399009	JX399040	JX399073
P. dracontomelon	MFLU 14–0207*	*Dracontomelon dao*	Anacardiaceae	Thailand	-	-	-	KP781880
P. ericacearum	IFRDCC 2439*	*Rhododendron delavayi*	Ericaceae	China	-	KC537807	KC537821	KC537814
P. furcata	**LC6303 = LF1310**	*Camellia* sp., endophyte	Theaceae	China, Yunnan, Xishuangbanna, Tian Ba	KX895130	KX895016	KX895349	KX895235
	**LC6601 = LF1245**	*C. sinensis*, pathogen	Theaceae	China, Yunnan, Xishuangbanna, Gua Feng Zhai	KX895133	KX895024	KX895357	KX895243
	**LC6691 = LF1371**	*C. sinensis*, pathogen	Theaceae	China, Yunnan, Xishuangbanna, Huang Zhu Peng	KX895136	KX895030	KX895363	KX895248
	MFLUCC 12-0054*	*Camellia sinensis*	Theaceae	Thailand	KM116283	JQ683724	JQ683708	JQ683740
P. gaultheria	IFRD 411-014*	*Gaultheria forrestii*	Ericaceae	China	-	KC537805	KC537819	KC537812
P. grevilleae	CBS 114127*	*Grevillea* sp.	Proteaceae	Australia	KM116212	KM199300	KM199407	KM199504
P. hawaiiensis	CBS 114491*	*Leucospermum* sp.	Myrtaceae	Hawaii	KM116239	KM199339	KM199428	KM199514
P. hollandica	CBS 265.33*	*Sciadopitys verticillata*	Sciadopityaceae	Netherlands	KM116228	KM199328	KM199388	KM199481
P. humus	CBS 115450	*Ilex cinerea*	Aquifoliaceae	Hong Kong	KM116208	KM199319	KM199418	KM199487

	CBS 336.97*	soil	-	Papua New Guinea	KM116230	KM199317	KM199420	KM199484
P. inflexa	MFLUCC 12-0270*	unidentified tree	-	China	-	JX399008	JX399039	JX399072
P. intermedia	MFLUCC 12-0259*	unidentified tree	-	China	-	JX398993	JX399028	JX399059
P. italiana	MFLU 14–0214*	*Cupressus glabra*	Cupressaceae	Italy	-	KP781878	KP781882	KP781881
P. jesteri	CBS 109350*	*Fragraea bodenii*	Gentianaceae	Papua New Guinea	KM116281	KM199380	KM199468	KM199554
P. jiangxiensis	**LC4242 = YH89**	*Eurya* sp., pathogen	Theaceae	China, Jiangxi, Lushan National Park	-	KX895035	KX895327	KX895213
	**LC4399 = YH257***	*Camellia* sp., pathogen	Theaceae	China, Jiangxi, Lushan National Park	KX895128	KX895009	KX895341	KX895227
P. jinchanghensis	**LC6636 = LF1281***	*C. sinensis*, pathogen	Theaceae	China, Yunnan, Xishuangbanna, Jin Chang He	KX895135	KX895028	KX895361	KX895247
	LC8190 = LF2080	*C. sinensis*, pathogen	Theaceae	China, Yunnan, Xishuangbanna, Jin Chang He	-	KY464144	KY464164	KY464154
	LC8191 = LF2081	*C. sinensis*, pathogen	Theaceae	China, Yunnan, Xishuangbanna, Jin Chang He	-	KY464145	KY464165	KY464155
P. kenyana	**LC3291 = LF513**	*C. sinensis*, pathogen	Theaceae	China, Jiangxi, Yangling	KX895093	KX894962	KX895294	KX895179
	**LC3633 = LF862**	*Camellia* sp., pathogen	Theaceae	China, Jiangxi, Ganzhou	KX895115	KX894992	KX895323	KX895209
	**LC6633 = LF1278**	*C. sinensis*, pathogen	Theaceae	China, Yunnan, Xishuangbanna, Jin Chang He	KX895134	KX895027	KX895360	KX895246
	CBS 442.67*	*Coffea* sp.	Rubiaceae	Kenya	KM116234	KM199302	KM199395	KM199502
	CBS 911.96	raw material from agar-agar	-	-	KM116204	KM199303	KM199396	KM199503
P. keteleeria	MFLUCC 13-0915*	*Keteleeria pubescens*	Pinaceae	China	-	KJ023087	KJ023088	KJ023089
P. knightia	CBS 111963	*Knightia* sp.	Proteaceae	New Zealand	KM116241	KM199311	KM199406	KM199495
	CBS 114138*	*Knightia* sp.	Proteaceae	New Zealand	KM116227	KM199310	KM199408	KM199497
P. licualacola	HGUP 4057*	*Licuala grandis*	Arecaceae	China	-	KC492509	KC481683	KC481684
P. linearis	MFLUCC 12-0271*	*Trachelospermum* sp.	Apocynaceae	China	-	JX398992	JX399027	JX399058
P. longiappendiculata	**LC3013 = LF221***	*C. sinensis*, pathogen	Theaceae	China, Fujian, Zhangzhou	-	KX894939	KX895271	KX895156
P. lushanensis	**LC4344 = YH198***	*Camellia* sp., pathogen	Theaceae	China, Jiangxi Province, Lushan National Park	KX895127	KX895005	KX895337	KX895223
	LC8182 = LF2072	*Camellia* sp., pathogen	Theaceae	China, Jiangxi Province, Lushan National Park	-	KY464136	KY464156	KY464146
	LC8183 = LF2073	*Camellia* sp., pathogen	Theaceae	China, Jiangxi Province, Lushan National Park	-	KY464137	KY464157	KY464147
P. macadamiae	BRIP 63738b*	*Macadamia integrifolia*	Proteaceae	Australia, New South Wales	-	KX186588	KX186680	KX186621

	BRIP 63739a	*Macadamia integrifolia*	Proteaceae	Australia, New South Wales	-	KX186589	KX186681	KX186622
	BRIP 63739b	*Macadamia integrifolia*	Proteaceae	Australia, New South Wales	-	KX186587	KX186679	KX186620
	BRIP 63741a	*Macadamia integrifolia*	Proteaceae	Australia, New South Wales	-	KX186586	KX186678	KX186619
P. malayana	CBS 102220*	*Macaranga triloba*	Euphorbiaceae	Malaysia	KM116238	KM199306	KM199411	KM199482
P. monochaeta	CBS 144.97*	*Quercus robur*	Fagaceae	Netherlands	KM116229	KM199327	KM199386	KM199479
	CBS 440.83	*Taxus baccata*	Taxaceae	Netherlands	KM116196	KM199329	KM199387	KM199480
P. novaehollandiae	CBS 130973*	*Banksia grandis*	Proteaceae	Australia	KM116232	KM199337	KM199425	KM199511
P. oryza	CBS 111522	*Telopea* sp.	Proteaceae	USA	-	KM199294	KM199394	KM199493
	CBS 171.26	-	-	Italy	KM116206	KM199304	KM199397	KM199494
	CBS 353.69*	*Oryza sativa*	Poaceae	Denmark	KM116221	KM199299	KM199398	KM199496
P. papuana	CBS 331.96*	coastal soil	-	Papua New Guinea	KM116240	KM199321	KM199413	KM199491
	CBS 887.96	*Cocos nucifera*	Arecaceae	Papua New Guinea	KM116231	KM199318	KM199415	KM199492
P. parva	CBS 265.37*	*Delonix regia*	Fabaceae	-	KM116226	KM199312	KM199404	KM199508
	CBS 278.35	*Leucothoe fontanesiana*	Ericaceae	-	KM116205	KM199313	KM199405	KM199509
P. portugalica	**LC0670 = ICMP13999**	*C. japonica*, pathogen	Theaceae	New Zealand, Mt Albert, AK	KX895066	KX894920	KX895252	KX895139
	**LC2929 = LF137**	*Camellia* sp., pathogen	Theaceae	Chengdu, Sichuan	KX895067	KX894921	KX895253	KX895138
	**LC2934 = LF142**	*Camellia* sp., pathogen	Theaceae	Chengdu, Sichuan	KX895068	KX894922	KX895254	KX895140
	**LC2935 = LF143**	*Camellia* sp., pathogen	Theaceae	Chengdu, Sichuan	KX895069	KX894923	KX895255	KX895141
	**LC2948 = LF156**	*Camellia* sp., pathogen	Theaceae	Chengdu, Sichuan	KX895048	KX894925	KX895257	KX895143
	**LC2949 = LF157**	*Camellia* sp., pathogen	Theaceae	Chengdu, Sichuan	KX895049	KX894926	KX895258	KX895144
	**LC2953 = LF161**	*Camellia* sp., pathogen	Theaceae	Chengdu, Sichuan	KX895070	KX894927	KX895259	KX895145
	**LC2954 = LF162**	*Camellia* sp., pathogen	Theaceae	Chengdu, Sichuan	KX895071	KX894928	KX895260	KX895146
	**LC2961 = LF169**	*Camellia* sp., pathogen	Theaceae	Chengdu, Sichuan	KX895072	KX894929	KX895261	KX895147
	**LC2984 = LF192**	*Camellia* sp., endophyte	Theaceae	Chengdu, Sichuan	KX895073	KX894930	KX895262	KX895148
	**LC3603 = LF832**	*Camellia* sp., pathogen	Theaceae	China, Jiangxi, Lushan Botanical Garden	KX895110	KX894987	KX895318	KX895204

	**LC3605 = LF834**	*Camellia* sp., pathogen	Theaceae	China, Jiangxi, Lushan Botanical Garden	KX895111	KX894988	KX895319	KX895205
	**LC4254 = YH103**	*Camellia* sp., pathogen	Theaceae	China, Jiangxi, Lushan National Park	KX895118	KX894996	KX895328	KX895214
	**LC4255 = YH104**	*Camellia* sp., pathogen	Theaceae	China, Jiangxi, Lushan National Park	KX895119	KX894997	KX895329	KX895215
	**LC4256 = YH105**	*Camellia* sp., pathogen	Theaceae	China, Jiangxi, Lushan National Park	KX895120	KX894998	KX895330	KX895216
	**LC4257 = YH106**	*Camellia* sp., pathogen	Theaceae	China, Jiangxi, Lushan National Park	KX895121	KX894999	KX895331	KX895217
	**LC4312 = YH162**	*Camellia* sp., pathogen	Theaceae	China, Jiangxi, Lushan National Park	KX895122	KX895000	KX895332	KX895218
	**LC4324 = YH176**	*C. chekiangoleosa*, pathogen	Theaceae	China, Jiangxi, Lushan National Park	KX895123	KX895001	KX895333	KX895219
	**LC4330 = YH183**	*Camellia* sp., pathogen	Theaceae	China, Jiangxi, Lushan National Park	KX895124	KX895002	KX895334	KX895220
	**LC4337 = YH191**	*Camellia* sp., pathogen	Theaceae	China, Jiangxi, Lushan National Park	KX895125	KX895003	KX895335	KX895221
	**LC4343 = YH197**	*Camellia* sp., pathogen	Theaceae	China, Jiangxi, Lushan National Park	KX895126	KX895004	KX895336	KX895222
	**LC4360 = YH216**	*C. japonica*, pathogen	Theaceae	China, Jiangxi, Lushan National Park	-	KX895006	KX895338	KX895224
	**LC4369 = YH225**	*Camellia* sp., pathogen	Theaceae	China, Jiangxi, Lushan National Park	-	KX895007	KX895339	KX895225
	**LC4370 = YH226**	*Camellia* sp., pathogen	Theaceae	China, Jiangxi, Lushan National Park	-	KX895008	KX895340	KX895226
	CBS 393.48*	-	-	Portugal	KM116233	KM199335	KM199422	KM199510
P. rhododendri	IFRDCC 2399*	*Rhododendron sinogrande*	Ericaceae	China	-	KC537804	KC537818	KC537811
P. rhodomyrtus	HGUP4230	*Rhodomyrtus tomentosa*	Myrtaceae	China	-	KF412648	KF412642	KF412645
	**LC3413 = LF635**	*C. sinensis*, pathogen	Theaceae	China, Jiangxi, Nanchang, Mei Ling	KX895109	KX894981	KX895313	KX895198
	**LC4458 = YH318**	*C. sinensis*, pathogen	Theaceae	Chian, Jiangxi, Yangling	-	KX895010	KX895342	KX895228
P. rosea	MFLUCC 12-0258*	*Pinus* sp.	Pinaceae	China	-	JX399005	JX399036	JX399069
P. scoparia	CBS 176.25*	*Chamaecyparis* sp.	Cupressaceae	-	KM116216	KM199330	KM199393	KM199478
P. sequoiae	MFLUCC 13-0399*	*Sequoia sempervirens*	Cupressaceae	Italy, Province of Forl ì-Cesena	KF572344	KX572339	-	-
Pestalotiopsis sp. 1	**LC3637 = LF866**	*Camellia* sp., pathogen	Theaceae	China, Jiangxi, Ganzhou	KX895116	KX894993	KX895324	KX895210
	**LC3640 = LF869**	*Camellia* sp., pathogen	Theaceae	China, Jiangxi, Ganzhou	KX895117	KX894995	KX895326	KX895212
Pestalotiopsis sp. 2	**LC3616 = LF845**	*Camellia* sp., pathogen	Theaceae	China, Jiangxi, Lushan Botanical Garden	KX895113	KX894990	KX895321	KX895207
Pestalotiopsis sp. 3	**LC6576 = LF1218**	*C. sinensis*, pathogen	Theaceae	China, Yunnan, Xishuangbanna, Gua Feng Zhai	KX895131	KX895021	KX895354	KX895240

	**LC6577 = LF1219**	*C. sinensis*, pathogen	Theaceae	China, Yunnan, Xishuangbanna, Gua Feng Zhai	KX895132	KX895022	KX895355	KX895241
	**LC6578 = LF1220**	*C. sinensis*, pathogen	Theaceae	China, Yunnan, Xishuangbanna, Gua Feng Zhai	KX895038	KX895023	KX895356	KX895242
Pestalotiopsis sp. Clade 33	CBS 263.33	*Rhododendron ponticum*	Ericaceae	Netherlands	KM116198	KM199316	KM199414	KM199489
	CBS 264.33	*Cocos* sp.	Arecaceae	Sulawesi	KM116199	KM199322	KM199412	KM199490
P. spathulata	CBS 356.86*	*Gevuina avellana*	Proteaceae	Chile	KM116236	KM199338	KM199423	KM199513
P. telopeae	CBS 114137	*Protea* sp., Pink Ice	Proteaceae	Australia	KM116219	KM199301	KM199469	KM199559
	CBS 114161*	*Telopea* sp.	Proteaceae	Australia	-	KM199296	KM199403	KM199500
	CBS 113606	*Telopea* sp.	Proteaceae	Australia	KM116202	KM199295	KM199402	KM199498
P. trachicarpicola	IFRDCC 2403	*Podocarpus macrophyllus*	Podocarpaceae	China	-	KC537809	KC537823	KC537816
	**LC4523 = YH388**	*C. sinensis*, pathogen	Theaceae	Chian, Jiangxi, Yangling	-	KX895011	KX895344	KX895230
	MFLUCC 12-0264	*Chrysophyllum* sp.	Sapotaceae	China	-	JX399004	JX399035	JX399068
	MFLUCC 12-0265	*Schima* sp.	Theaceae	China	-	JX399003	JX399034	JX399067
	MFLUCC 12-0266	*Sympolocos* sp.	Symplocaceae	China	-	JX399002	JX399033	JX399066
	MFLUCC 12-0267	unidentified tree	-	China	-	JX399001	JX399032	JX399065
	OP068*	*Trachycarpus fortunei*	Arecaceae	China	-	JQ845947	JQ845945	JQ845946
	MFLUCC 12-0263	unidentified tree	-	China	-	JX399000	JX399031	JX399064
P. unicolor	MFLUCC 12-0276*	*Rhododendron* sp.	Ericaceae	China	-	JX398999	JX399030	-
	MFLUCC 12-0275	unidentified tree	-	China	-	JX398998	JX399029	JX399063
P. verruculosa	MFLUCC 12-0274*	*Rhododendron* sp.	Ericaceae	China	-	JX398996	-	JX399061
P. yanglingensis	**LC3067 = LF275**	*C. sinensis*, pathogen	Theaceae	Chian, Jiangxi, Yangling	KX895080	KX894949	KX895281	KX895166
	**LC3068 = LF276**	*C. sinensis*, pathogen	Theaceae	Chian, Jiangxi, Yangling	KX895081	KX894950	KX895282	KX895167
	**LC3152 = LF361**	*C. sinensis*, pathogen	Theaceae	Chian, Jiangxi, Yangling	KX895088	KX894957	KX895289	KX895174
	**LC3153 = LF362**	*C. sinensis*, pathogen	Theaceae	Chian, Jiangxi, Yangling	KX895089	KX894958	KX895290	KX895175
	**LC3321 = LF543**	*C. sinensis*, pathogen	Theaceae	Chian, Jiangxi, Yangling	KX895095	KX894965	KX895297	KX895182

	**LC3331 = LF553**	*C. sinensis*, pathogen	Theaceae	Chian, Jiangxi, Yangling	KX895097	KX894967	KX895299	KX895184
	**LC3365 = LF587**	*C. sinensis*, pathogen	Theaceae	Chian, Jiangxi, Yangling	KX895058	KX894972	KX895304	KX895189
	**LC3372 = LF594**	*C. sinensis*, pathogen	Theaceae	Chian, Jiangxi, Yangling	KX895102	KX894973	KX895305	KX895190
	**LC3373 = LF595**	*C. sinensis*, pathogen	Theaceae	Chian, Jiangxi, Yangling	KX895103	KX894974	KX895306	KX895191
	**LC3375 = LF597**	*C. sinensis*, pathogen	Theaceae	Chian, Jiangxi, Yangling	KX895104	KX894975	KX895307	KX895192
	**LC3377 = LF599**	*C. sinensis*, pathogen	Theaceae	Chian, Jiangxi, Yangling	-	KX894977	KX895309	KX895194
	**LC3396 = LF618**	*C. sinensis*, pathogen	Theaceae	Chian, Jiangxi, Yangling	KX895106	KX894978	KX895310	KX895195
	**LC3397 = LF619**	*C. sinensis*, pathogen	Theaceae	Chian, Jiangxi, Yangling	KX895107	KX894979	KX895311	KX895196
	**LC3412 = LF634**	*C. sinensis*, pathogen	Theaceae	China, Jiangxi, Nanchang, Mei Ling	KX895108	KX894980	KX895312	KX895197
	**LC4553 = YH420***	*C. sinensis*, pathogen	Theaceae	Chian, Jiangxi, Yangling	-	KX895012	KX895345	KX895231
P. yunnanensis	HMAS 96359*	*Podocarpus macrophyllus*	Podocarpaceae	China	-	AY373375	-	-
Ps. ampullacea	**LC4479**	Lauraceae, pathogen	Lauraceae	Chian, Jiangxi	-	KX895034	KX895343	KX895229
	**LC6618 = LF1263***	*C. sinensis*, pathogen	Theaceae	China, Yunnan, Xishuangbanna, Jing Mai	KX895039	KX895025	KX895358	KX895244
Ps. camelliae-sinensis	CGMCC 3.9188	*Camellia sinensis*	Theaceae	China	-	JN943624	JQ683704	JQ683736
	CGMCC 3.9192	*Camellia sinensis*	Theaceae	China	-	JN943622	KU562851	KU562850
	**LC3009 = LF217**	*C. sinensis*, pathogen	Theaceae	China, Fujian, Zhangzhou	KX895050	KX894935	KX895267	KX895152
	**LC3010 = LF218**	*C. sinensis*, pathogen	Theaceae	China, Fujian, Zhangzhou	KX895051	KX894936	KX895268	KX895153
	**LC3020 = LF228**	*C. sinensis*, pathogen	Theaceae	China, Fujian, Zhangzhou	KX895054	KX894940	KX895272	KX895157
	**LC3021 = LF229**	*C. sinensis*, pathogen	Theaceae	China, Fujian, Zhangzhou	-	KX894941	KX895273	KX895158
	**LC3022 = LF230**	*C. sinensis*, pathogen	Theaceae	China, Fujian, Zhangzhou	KX895055	KX894942	KX895274	KX895159
	**LC3023 = LF231**	*C. sinensis*, pathogen	Theaceae	China, Fujian, Zhangzhou	KX895056	KX894943	KX895275	KX895160
	**LC3487 = LF714**	*C. sinensis*, pathogen	Theaceae	China, Guilin	KX895061	KX894984	KX895315	KX895201
	**LC3490 = LF718***	*C. sinensis*, pathogen	Theaceae	China, Guilin	KX895062	KX894985	KX895316	KX895202
	**LC3571 = LF799**	*C. sinensis*, pathogen	Theaceae	China, Hangzhou	KX895063	KX894986	KX895317	KX895203
	**LC6677 = LF1357**	*C. sinensis*, pathogen	Theaceae	China, Yunnan, Xishuangbanna, Da Zhai	KX895044	KX895029	KX895362	-

Ps. chinensis	**LC3011 = LF219***	*C. sinensis*, pathogen	Theaceae	China, Fujian, Zhangzhou	KX895052	KX894937	KX895269	KX895154
	**LC3012 = LF220**	*C. sinensis*, pathogen	Theaceae	China, Fujian, Zhangzhou	KX895053	KX894938	KX895270	KX895155
	**LC6306 = LF1313**	*C. sinensis*, endophyte	Theaceae	China, Yunnan, Xishuangbanna, Tian Ba	KX895043	KX895017	KX895350	KX895236
	**LC6629 = LF1274**	*C. sinensis*, pathogen	Theaceae	China, Yunnan, Xishuangbanna, Man Nai	KX895040	KX895026	KX895359	KX895245
	**LC6695 = LF1375**	*C. sinensis*, pathogen	Theaceae	China, Yunnan, Xishuangbanna, Gao Shan	KX895045	KX895031	KX895364	KX895249
	**LC6711 = LF1391**	*C. sinensis*, pathogen	Theaceae	China, Yunnan, Xishuangbanna, Lao Man Sa	KX895046	KX895032	KX895365	KX895250
Ps. cocos	CBS 272.29*	*Cocos nucifera*	Arecaceae	Java	KM116276	KM199378	KM199467	KM199553
Ps. ignota	NN 42909*					KU500020		KU500016
Ps. indica	CBS 459.78*	*Hibiscus rosa-sinensis*	Malvaceae	India	-	KM199381	KM199470	KM199560
Ps. kubahensis	UMAS KUB-P20*	*Macaranga* sp.	Chamaesyce	Kubah	-	KT006749	-	-
Ps. simitheae	MFLUCC 12-0121*	*Pandanus odoratissimus*		Thailand		KJ503812	KJ503815	KJ503818
	MFLUCC 12-0125	*Pandanus odoratissimus*		Thailand		KJ503813	KJ503816	KJ503819
Ps. theae	SC011	*Camellia sinensis*	Theaceae	Thailand	-	JQ683726	JQ683710	JQ683742
	MFLUCC 12-0055*	*Camellia sinensis*	Theaceae	Thailand	KM116282	JQ683727	JQ683711	JQ683743
